# Utilisation of Chick Embryo Chorioallantoic Membrane as a Model Platform for Imaging-Navigated Biomedical Research

**DOI:** 10.3390/cells10020463

**Published:** 2021-02-22

**Authors:** Lei Chen, Shuncong Wang, Yuanbo Feng, Jinyong Zhang, Yuqing Du, Jiang Zhang, Chantal Van Ongeval, Yicheng Ni, Yue Li

**Affiliations:** 1 KU Leuven, Biomedical Group, Campus Gasthuisberg, 3000 Leuven, Belgium; lei.chen@kuleuven.be (L.C.); shuncong.wang@kuleuven.be (S.W.); yuanbo.feng@kuleuven.be (Y.F.); chantal.vanongeval@uzleuven.be (C.V.O.); 2 Shanghai Key Laboratory of Molecular Imaging, Shanghai University of Medicine and Health Sciences, Shanghai 201318, China; 192672234@st.usst.edu.cn (J.Z.); luojy@sumhs.edu.cn (Y.D.); 3 School of Medical Instrument and Food Engineering, University of Shanghai for Science & Technology, Shanghai 200093, China; 4 Faculty of Agricultural Biotechnology and Ecotechnology, Shanghai Vocational College of Agriculture and Forestry, Shanghai 201600, China; zhangj@shafc.edu.cn

**Keywords:** chick embryo, chorioallantoic membrane (CAM), preclinical pharmacological research, vascular disrupting agents (VDAs), imaging techniques

## Abstract

The fertilised chick egg and particularly its chorioallantoic membrane (CAM) have drawn continuing interest in biomedicine and bioengineering fields, especially for research on vascular study, cancer, drug screening and development, cell factors, stem cells, etc. This literature review systemically introduces the CAM’s structural evolution, functions, vascular features and the circulation system, and cell regulatory factors. It also presents the major and updated applications of the CAM in assays for pharmacokinetics and biodistribution, drug efficacy and toxicology testing/screening in preclinical pharmacological research. The time course of CAM applications for different assays and their advantages and limitations are summarised. Among these applications, two aspects are emphasised: (1) potential utility of the CAM for preclinical studies on vascular-disrupting agents (VDAs), promising for anti-cancer vascular-targeted therapy, and (2) modern imaging technologies, including modalities and their applications for real-time visualisation, monitoring and evaluation of the changes in CAM vasculature as well as the interactions occurring after introducing the tested medical, pharmaceutical and biological agents into the system. The aim of this article is to help those working in the biomedical field to familiarise themselves with the chick embryo CAM as an alternative platform and to utilise it to design and optimise experimental settings for their specific research topics.

## 1. Introduction

To produce new medications which are safe and efficacious, and can pass all regulatory requirements, pharmaceutical development must proceed through several stages: (1) drug candidate discovery, (2) molecule characterisation, (3) formulation for delivery, (4) pharmacokinetics and biodistribution, (5) efficacy and toxicology testing, (6) investigational new drug (IND) application, (7) bioanalytical testing and (8) phase I–IV clinical trials. The intermediate in vivo preclinical studies of the above stages 4 and 5 are traditionally conducted, by the laboratories of academia or industry, on experimental animals without and with human disease simulation. However, animal welfare and protection are raising more and more public concerns. The currently advocated guiding policy is to replace, reduce, and refine (3 Rs) animal use in research. Therefore, with plausible ethical, scientific, legal and economic reasons, it is necessary to develop scientific methods or platforms to reduce the need for animals or eventually to replace them entirely in scientific research and the pharmaceutical industry. Thus, fertilised eggs or chick embryos have become a good choice.

The egg embryo is considered a good pharmaceutical testing platform with the following advantageous features: (1) the egg embryo is a complete creature, with necessary organs within an isolated environment; (2) the size of the egg embryo is small and easy to handle; (3) the egg embryo contains rich nutrients and vigorous angiogenesis capacities; (4) eggs do not possess a complete immune system at certain stages of chick development and are much less expensive than immune-compromised animals; and (5) eggs are less restricted with animal welfare concerns because they are not considered animals yet.

Embryologically, during the development of fertilised eggs, an extremely rich vascular network is generated between the double layers of the chorioallantoic membrane (CAM). This vascular network fuses closely underneath the eggshell and connects to the embryonic circulation via the allantoic stalk. The CAM system has been widely used in in vivo assays for studying angiogenesis [1] and for human tumour growth and therapies [2]. Indeed, angiogenesis and anti-angiogenesis processes related to tissues, cells or soluble factors are tested by the CAM [2]. Many substances have been reported to boost or inhibit angiogenesis in the CAM, for instance growth factors, anti-cancer agents, pro-angiogenic molecules, natural and synthetic molecules, antibodies, organic-metallic compounds, antibiotics, etc. [3].

Meanwhile, the classical property of the CAM with a native vasculature already allows testing the pharmacological effects of certain compounds in biomedical research. Small-molecule vascular disrupting agents (VDAs) constitute a new therapy for cancer and can selectively affect the tumour vasculature via some pathways to inhibit blood flow and cause extensive necrosis within the tumour [3,4,5]. Therefore, VDAs are ideal candidates for demonstration of the efficiency and feasibility of the CAM platform in preclinical efficacy and toxicology testing of pharmaceuticals. To the best of our knowledge, only few studies have been conducted in this area.

The crucial step in evaluation is to observe and monitor vascular alterations, including angiogenesis, vascular disrupting processes following administration of tested medicines, etc. Researchers have developed different techniques to measure and quantify these properties in the CAM of the chick embryo. The techniques can be classified into destructive (ex ovo) and non-destructive (in ovo) and qualitative, semi-quantitative and quantitative ones [6,7].

Here, we systematically review existing CAM assays for different applications and the available techniques for measuring and monitoring the vasculature in the CAM. Some new imaging techniques are emphasised, which permit us to visualise the vascular structure at the microscopic level. Moreover, new techniques permit us to continuously and dynamically visualise and record the changes in the vasculature and carry out quantified analysis and assessment. Modern techniques and methods can save time, materials, and labour, with reduced experimental deviations. 

This review outlines the potential of the CAM platform to evaluate the pharmacological effects of VDAs, from the most advanced imaging instrument for vasculature change measurements.

## 2. CAM Assay

### 2.1. Development of Fertilised Chick Eggs in Incubation

**Figure 1:**
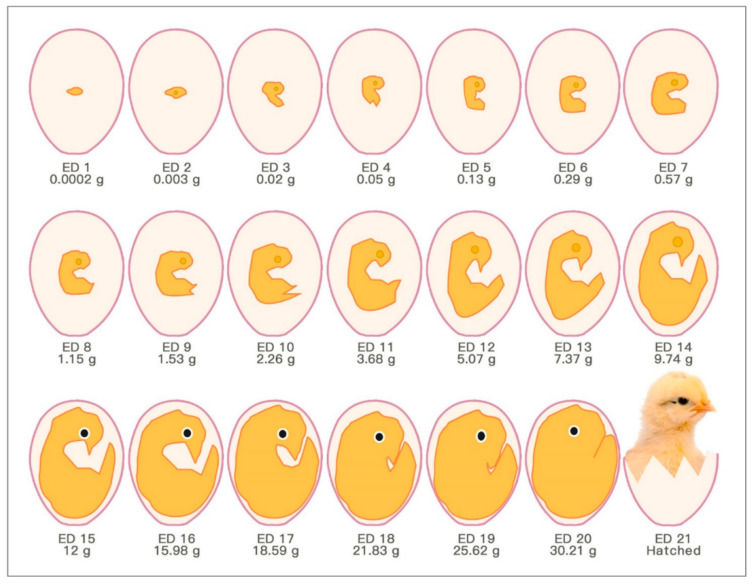
Schematic drawings of chronological chick embryo development.

The chick embryo normally experiences 21 days of development before hatching, which corresponds to multiple stages (Figure 1). Usually, the first day of incubation is defined as embryonic day one (ED 1) [8].

**Figure 2:**
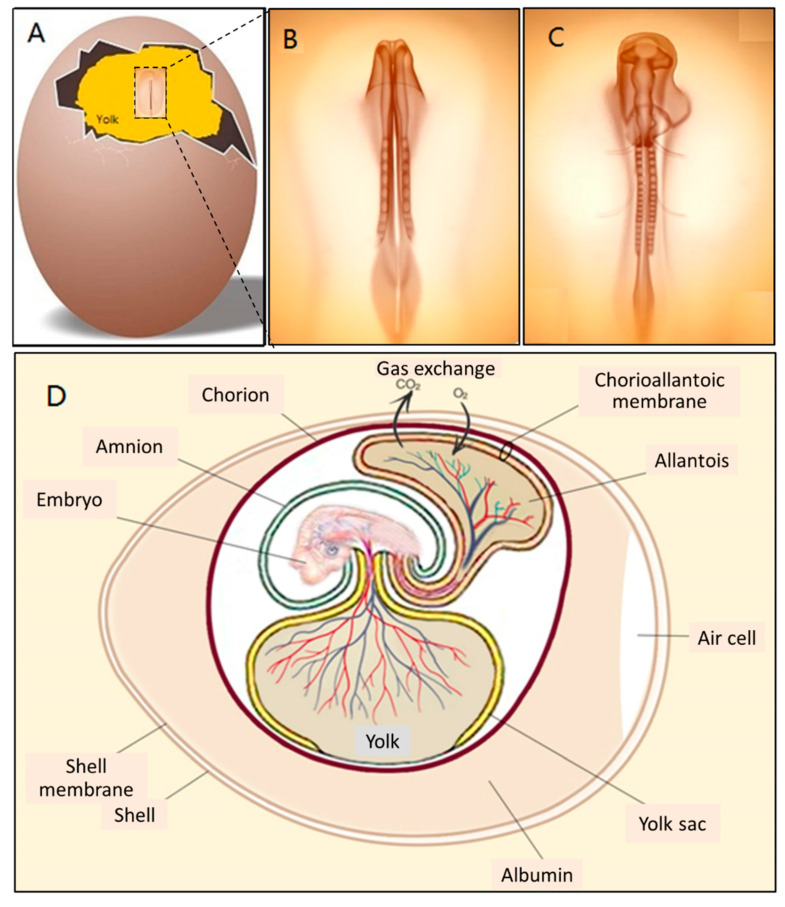
(**A**) A blastoderm floating on the surface of yolk on ED 2, (**B**,**C**) magnified views of the blastoderm in development on ED 2 and (**D**) illustration of membranes and blood circulation system of an embryonic chick egg in the middle stage.

In fact, embryonic development starts in the chick egg before ED 1. The process of blastoderm generation was described long ago [9]. After mating, the female ovum meets with the male sperm cell to form a fertilised cell. Cell divisions initiate around 5 h after insemination, and then a cluster of cells is generated on the surface of the yolk (Figure 2A). This cluster of cells is called the blastoderm, which grows into the embryo (Figure 2B,C). Later, the blastoderm continues to divide into three germ layers: ectoderm, mesoderm and endoderm.

Then, the blastoderm evolves into the embryo as follows: The mesoderm separates into two layers, upper (somatic mesoderm) and lower (splanchnic mesoderm). Spaces between the two layers are surrounded and form the coelom. The ectoderm and the somatic mesoderm compose the somatopleure; the endoderm and the splanchnic mesoderm compose the splanchnopleure. The coelom merges the embryo body with the middle parts of the somatopleure and splanchnopleure. 

The extra-embryonic parts grow into different membranes, including the yolk sac, the amnion, the chorion and the allantois. The chorion and the amnion are derived from the extra-embryonic somatopleure, and the yolk sac derives from the extra-embryonic splanchnopleure. The embryo body gets separated from the extra-embryonic tissues, only with the umbilicus connection. The endodermal and ectodermal tissue become epithelial cells of the membranes, and the mesodermal tissue creates the blood from and to this epithelium. 

The yolk sac is the membrane enclosing the yolk, and digested yolk can pass into the embryonic blood system through it [10].

The amnion is a sac surrounding the embryo. It can secrete liquid to cushion the embryo and keep it from dehydration [10].

Chorioallantois is the membrane generated at the final stage and is formed by the fusion of the chorion and the allantois. The allantois becomes a balloon-like shape outside the embryo body by ED 4 and begins to fuse with the inside of the chorion by EDs 6–7, forming the chorioallantoic membrane (CAM).

Three extra-embryonic membranes are there to support and nourish the embryo during growth: the yolk sac, the amnion and the CAM [11].

These membranes feature internal variable structures in eggs during embryo development. The size, morphology and position of the three membranes keep changing. All these changes are aimed to adapt the embryo development and physiological functions.

The extra-embryonic circulation has been distinguished from the intra-embryonic circulation [9]. The vitelline circulation and the allantoic circulation are extra-embryonic circulations. Major blood vessels connect different parts of the embryonic circulation (Figure 2D).

The circulating blood volume of the embryo from ED 4 until ED 18 has been determined (Table 1) [12]. The blood volume does not show an entire perfect curve of exponential growth. The blood volume reaches a peak value between ED 16 and ED 18 and decreases somewhat towards the end of hatching. This volume reduction is related to the degeneration of the extra-embryonic circulation system. 

Maina [13] comprehensively explained how the embryo absorbs vital nutrients from the albumen and the yolk to build new tissues and sustain existing organs during incubation before internal pipping (the chick pecks the CAM and the shell breaks). The calcium deposited in the eggshell provides the necessary source for bone formation in the chick embryo.

### 2.2. CAM Development and Physiological Function

**Figure 3:**
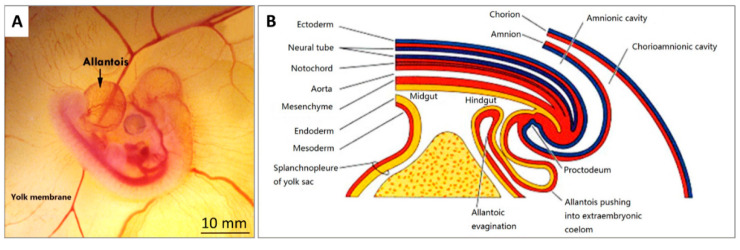
(**A**) A photo of a fertilised chick egg at day 4 post-incubation and (**B**) a schematic drawing displaying how the allantois is formed.

The CAM is a double-layer membrane fused by the chorion and the allantois and has an extremely rich vascular network. The vascular network in the CAM is connected with the embryonic circulation through the allantoic stalk (Figure 3A). The CAM is responsible for embryonic respiration, protecting and nourishing the embryo during most of the chick embryonic development. 

The formation of the allantois is shown in Figure 3B. The allantois of the embryo emerges at about 3.5 embryonic days (ED 3.5). The ventral wall of the endodermal hind gut of the embryo evaginates. This evagination pushes out a part of the embryo body into the extra-embryonic coelom. Its proximal portion (allantoic stalk) lies parallel and just next to the bottom of the yolk sac, and when the distal portion extends away from the embryo, it grows bigger. It is named the allantoic vesicle [14].

The allantoic vesicle enlarges very rapidly during EDs 4–10 [14]. As shown in Figure 3A, a chicken embryo at ED 4 forms the vascularised allantoic membrane. The background is the yolk sac membrane (YSM), also highly vascularised [15]. 

By the end of ED 7, most of the chorion is in contact with the shell membrane [16].

**Figure 4:**
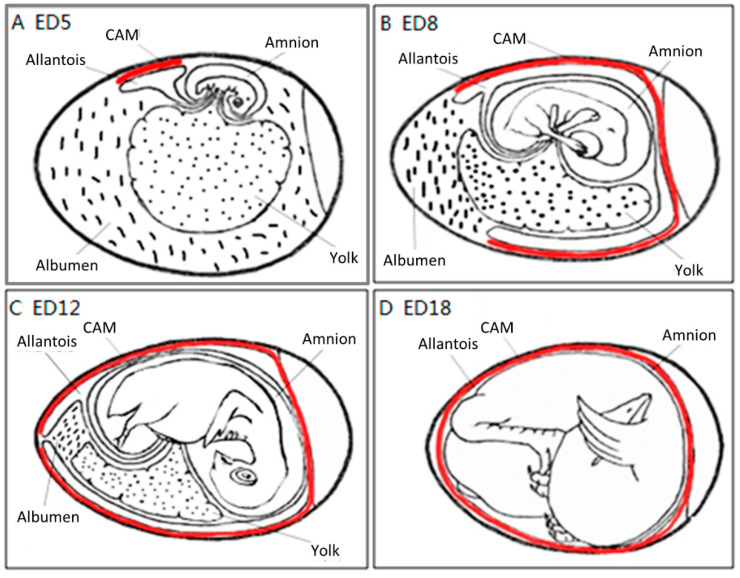
The development of the chorioallantoic membrane (CAM) (red lines) at different stages: (**A**) ED 5 initial coverage, (**B**) ED 8 half coverage, (**C**) ED 12 and (**D**) ED 18 full coverage of the chick embryo.

The process to form the CAM has been further described previously [9]. Through the continuous enlargement of the allantoic vesicle, at about 100 h of incubation, the allantois starts to fuse with the chorion, and consequently, the CAM is formed. The CAM consists of three layers (chorion, stroma, allantoic membrane) and lies close to the eggshell (Figure 4). 

The CAM spreads over the yolk sac surface and covers it completely between ED 6 and ED 7 [16]. This is consistent with Romanoff’s description [9]: around EDs 7–8, the CAM extends throughout the blunt half of the egg and reaches the middle line. He concluded that eventually, the CAM covers the entire egg around EDs 10–11.

As illustrated in Figure 4, the chorioallantois extends to embrace the contents of the whole egg at ED 12, attaching the entire surface of the inner shell membrane [17]. The CAM surface is 6 cm^2^ at ED 6, undergoing rapid extension, and becomes 65 cm^2^ at ED 14 [18].

Although the CAM becomes increasingly large, the two extra-embryonic circulations remain separate, but both are connected to the intra-embryonic circulation. 

**Figure 5:**
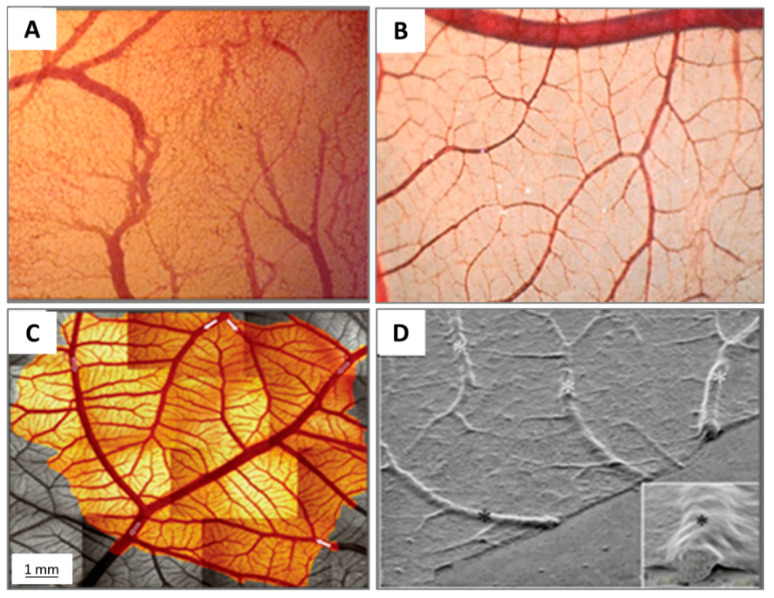
Images illustrating the maturation of the CAM vasculature. (**A**) Vascular remodelling, growth and anastomosis can be seen at ED 7; (**B**) hierarchic vascular structures and fully differentiated vessels are noted on ED 10; (**C**) vessel tree reconstruction and flow direction for major arterial and venous vessels (arrows); and (**D**) scanning electron microscope view of blood vessels on the CAM.

Coming to the end, apoptosis progresses in the CAM at ED 18, and apoptotic cells are found in the mesenchyme [8]. Finally, during internal piping, chick lung ventilation initiates CAM degeneration [19]. The outer and inner shell membranes interface each other with the eggshell, the chorion epithelium and the subchorionic sinuses (Figure 5), which brings capillary blood close to the air via the air cell [13]. 

The CAM is a highly vascularised and transparent membrane [11]. Arteries, veins and the capillary plexus exist in the CAM [2,20]. The chorioallantoic capillary volume and surface size increase during CAM development [18].

The CAM performs the gas-exchange function through the aerial vascular interface, when the chick embryo starts developing before ED 6 [9].

Since the CAM is subjacent to the inner shell membrane at ED 5, this highly vascularised membrane serves as the respiratory system of the embryo and is solely responsible for gas exchange until ED 19 [20]. Here is the mechanism of gas exchange: the respiratory exchange of oxygen and carbon dioxide is a passive diffusion process between the embryo and the environment, the eggshell and the CAM confer a resistance function, and the vasculature in the CAM is correlated with the O_2_ uptake ability of the embryo in this diffusion process. The general trend of this function is a gradual increase from ED 6 to EDs 14–15, and then a plateau phase [19,21,22]. The demand for O_2_ increases throughout embryo development, with increased production of metabolic end products [23].

Meanwhile, the allantois works as a deposit for waste products excreted by embryo kidneys, mostly urea at the early stage and chiefly uric acid at a later stage [9,14]. 

The CAM has other physiological functions: transportation of electrolytes (sodium and chloride) from the allantois and calcium movement from shell to bone for mineralisation. Calcium movement can be certified by typical calcium-transporting cells in the chorionic cavity by ED 12, and the movement rate can reach 100 nmol of calcium per hour per 1 cm^2^ of the CAM surface [8,14,24].

### 2.3. Microcirculation and Morphology in the CAM

A full understanding of the vascular structure of the CAM is essential to investigate the mechanisms of the vascular response in the CAM assay platform. 

The vascular system is the first system built up in the chick embryo in order to facilitate respiration, because oxygen molecules only can diffuse about 100–200 μm in embryonic tissues [25]. 

As shown in Figure 5, the CAM holds a rich vascular network within its mesodermal layer, and paired allantoic (umbilical) arteries and veins supply blood to this system [18].

**Figure 6:**
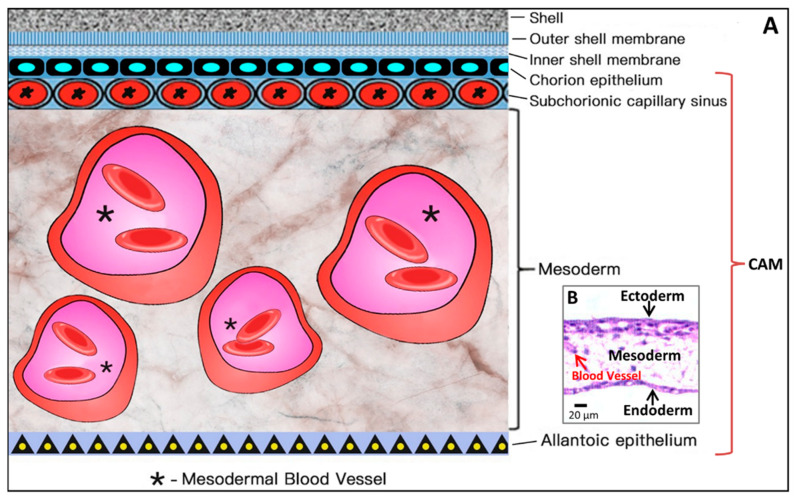
Simplified schematic diagram (**A**) showing the structural components of the CAM, which amplifies the embedded microscopic view of the haematoxylin-eosin-stained slice of the CAM (**B**) where the ectoderm includes the chorion epithelium and sub-chorionic capillary sinus layers of the CAM, and the endoderm corresponds to the allantoic epithelium of the CAM. *Larger vessels in the mesoderm (stroma).

The different stages of the vascular development process in the CAM present specific characteristics [26]. On ED 4, all vessels remain undifferentiated capillaries, whose walls only have a single endothelial layer without a basal lamina. By ED 8, primary vessels grow and differentiate into an artery–venous system and thus create a capillary network. These small, thin-walled capillaries with a 10–15 µm lumenal diameter migrate and present at the superficial layer of the CAM, which is just beneath the chorionic epithelium (Figure 6). 

Meanwhile, as the CAM is expanding, endothelium mitosis undergoes a rapid phase in the capillary network. Concurrently, the capillary endothelium in the CAM undergoes a sequence of structural changes [16]. A capillary network develops via respective angiogenic processes, including sprouting, elongation, fusion to the capillary plexus and intussusceptive growth [27]. The total length of the CAM vessels is closely correlated with the entire area of the CAM [27].

Other vessels located in the mesodermal layer are bigger generally, with a 10–115 µm diameter, and the endothelium of the blood vessels is surrounded by a layer of mesenchymal cells and completely wrapped by a basal lamina (Figure 6).

The capillary plexus is formed and develops quickly from EDs 4–5 by vasculogenesis and sprouting angiogenesis [28]. After short-term sprouting, intussusceptive growth remains dominant and increases density and complexity [27]. Intussusceptive growth is the main process from EDs 7–11, with lower endothelial cell proliferation [29], and the proliferation comes down after ED 11 [30]. Within this network, parts of the vessels become larger and extend laterally to form arteriolar and venular trees [27] (Figure 5C). EDs 10–12 is an important period for vascular development in the CAM. During EDs 10–12, the capillaries become adjacent to the chorionic epithelium (Figure 6).

Now, arteries and veins are distinguished in the mesodermal vessels. For example, a piece of 1.7 cm^2^ CAM contains two major arteries and one major vein. Diameters of the two main arterioles are 261 µm and 172 µm, and the major venule diameter is 390 µm [31]. Arteries have walls containing one or two layers of mesenchymal cells with more connective tissue surrounding the endothelium, tending to develop a fibroblast-type adventitia. Veins have walls surrounded by connective tissues and incomplete mesenchymal cells, which develop into smooth muscle cells [26]. CAM vessels grow rapidly up to ED 11, and then the endothelial cell mitosis dramatically reduces and stays at a minimal growth rate [26]. The density and fractal dimension of the vascular network increase steadily from EDs 6 to 14 and stop at ED 15, for both complexity and branching patterns [32,33].

The successive development of the CAM has been studied [31]. At ED 14, the capillary plexus invades and is located at the surface of the ectoderm underneath the shell membrane. The larger vessels in the mesoderm can float freely and move with the spontaneous movement of the embryo [16], whereas the capillary plexus is embedded in the most superficial layer of the CAM [15], as illustrated in Figure 6.

The CAM microcirculation is supplied by two primary arteries and drained by a single vein. These primary vessels connect several vessels from the pre- and post-capillaries of next generations [18]. The left and right allantoic arteries and vein remain apart during the fast growth of the CAM [31]. The interspace of major vessels allows inter-digitating arteriolar and venular trees to grow and let relatively few vessels cross.

In the assay platform to test the response of blood vessels, these large and primary vessels are the best target to be measured and evaluated in quantification by modern imaging techniques (medical and bioengineering). 

**Figure 7:**
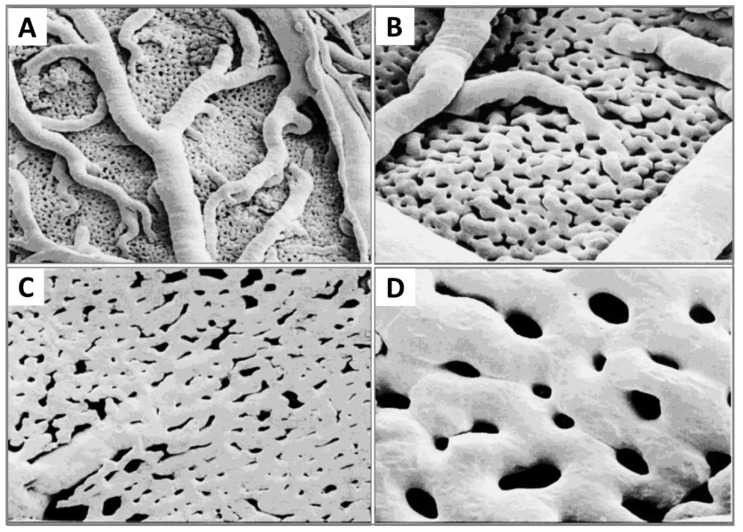
Resin corrosion cast (Mercox cast) of the developing CAM vasculature at ED 12. (**A**,**B**) A three-dimensional structure containing a capillary plexus and a layer of supplying and collecting vessels is recognisable. (**C**,**D**) Numerous pillars of different sizes caused by intussusceptive angiogenesis processes. Original magnification: (**A**) 3100, (**B**,**C**) 3200 and (**D**) 3400. Reproduced with permission from [11].

Now that the expansion of the CAM vasculature network is well established, branching patterns and microcirculation of pre- and post-capillaries of the CAM will be studied. The morphology and mechanism of such microcirculation are described in detail. The CAM consists of a superficial 2D network of very dense capillaries, named the capillary plexus, shown by scanning electron microscopy in Figure 7 as a mesh morphology [34]. The capillary mesh wraps a surrounding 3D space. The spatial configuration consists of two horizontal planes, which are connected by pre-capillary bridging vessels. These bridging vessels rise in an oblique-to-vertical direction towards the superficial capillary plane. The medium and large arterial and venous vessels, which supply and drain the superficial layer, are located within this 3D space [16].

The pre-capillary arterioles connect directly with the capillary network. However, the post-capillary venules connect with the superficial network via the venous sinus system. The small sinus is formed by the confluence of capillaries at the beginning of the post-capillary venules. The blood from the arterial tree streams into the superficial capillary mesh and then drains into the venous system. It is possible to distinguish the arterial and the venous blood in the circulatory system of the CAM by a smooth muscle layer surrounding the endothelium of the arterioles, but not that of the venules. Thus their morphology is different [16]. Another important conclusion is that blood vessels in the CAM have no terminal vessels, tips or sprouts and are always exhibited as a closed cycle [34].

More recently, morphology studies about microcirculation in the CAM have been deepened to discover how arterial and venous vessel trees integrate (connect) and communicate with the capillary bed [31,35]. These are described on a smaller scale by scanning electron microscopy [31]. The interdigitating pattern derives from arterial and venous vessel trees, and long arterial pathways link with short venous pathways through the capillary mesh, and vice versa. A distinct feature is the presence of new connections between arteriole and venule trees, which feed and drain the underlying plexus not only at their termini but also along proximal segments of the vessel tree.

### 2.4. Growth and Regulation Factors of Angiogenesis in CAM

The angiogenesis process is controlled by the balance of multiple growth factors which have proliferative and inhibitory regulatory activity [33]. A variety of growth factors have been revealed [11,14,36,37,38,39], which induce and promote CAM angiogenesis and undertake precise spatial and temporal regulations to form a mature vascular network (Table 2).

Vascular endothelial growth factor (VEGF) plays a leading role among these factors and is crucial for the angiogenic expansion at the early stage of CAM development. An endogenous VEGF-A presents two peaks at EDs 8–9 and 11–12 [40].

VEGF is the prime factor to attract migrating endothelial cells and stabilise vessels by bounding substrates during the formation of vascular tubes [36]. Some researchers believe that VEGF causes vascular permeability, recruits endothelial cells and inhibits vessel stabilisation. The fibroblast growth factor (FGF) family is the most potent cytokine to stimulate mitogenesis of multiple cell types, such as endothelial cells, osteoblasts, bone marrow stromal cells, mesenchymal stem cells and immune cells. It directly causes fibrogenesis [37]. At present, there is a general consensus that VEGF and FGF are main factors for the vascular growth regulation in the chick CAM.

FGF-1 and FGF-2 are major prototypic members of the FGF family. Both of them initiate activation immediately after binding to their cell surface receptor, which is named fibroblast growth factor receptor-1 (FGFR1), one of the receptor tyrosine kinases. FGF-1 is considered as a standard angiogenesis stimulator [39]. Endogenous FGF-2 may affect the proliferation, movement, redistribution and invasion of endothelial cells [11]. FGF-2 becomes detectable in the CAM since ED 6, and maximal concentrations occur between ED 10 and ED 14. Meanwhile, FGF located in the CAM can regulate angiogenesis [38].

However, VEGF and FGF are not sufficient to finish angiogenesis, because they function not only as promoters of endothelial cell proliferation but also as inhibitors of vessel maturation. Through suppressing receptors on smooth muscle cells, VEGF inhibits pericyte coverage of vascular sprouts and renders existing vessels unstable, whereas platelet-derived growth factor (PDGF) creates vascular stability at the maturation stage (late stage) of angiogenesis [37].

PDGF drives pericytes and smooth muscle cells to recruit, which form a layer of cells around new capillaries, embed the endothelial lining and facilitate binding strongly to the extracellular matrix. These are necessary conditions to stabilise new blood vessels. Meanwhile, PDGF is functional to inhibit the recruitment of endothelial cells.

These facts show that a single factor is not sufficient to create a stable mature vasculature. However, the coordination and balance of multiple factors can induce a successful angiogenic response and mature vessel network in the CAM. 

Not many concrete studies have been carried out on angiopoietins (ANGs). ANGs play an important role in endothelial sprouting, mural cell recruiting and vessel wall remodelling [36]. Angiopoietin-1 (ANG-1), a 498-amino-acid glycoprotein, is a ligand of the endothelium-specific receptor Tie2 [41]. It recruits periendothelial cells, at the late stage of vascular maturation, in the presence of VEGF. Unlike VEGF, ANG-1 has no mitogenic effect in vivo [42]. However, another report indicated that ANG-2 can induce rapid initiation of blood vessels [37].

Hepatocyte growth factor (HGF), a specific growth factor for the liver, shows the highest expression at the beginning of chick embryo development. Meanwhile, HGF is a cytokine which stimulates the proliferation and morphogenesis of epithelia [36]. HGF can also directly act on endothelial cells, including stimulation of proliferation, cell mobility, protease production and organisation of capillary-like tubes [43].

Hypoxia-inducible factor 1-alpha and 2-alpha (HIF-1α and HIF-2α) are activated by a hypoxic environment. HIF may switch on the expression of angiogenic genes, for example VEGF, which attract branching vessels to stretch towards hypoxic tissues [36,44]. A high expression level of HIF-1α correlates with a peak in the angiogenic process in the CAM [14].

Among anti-angiogenic molecules, endostatin, a proteolytic fragment of collagen XVIII (a component of the basement membrane), inhibits endothelial cell survival and migration. During angiogenesis in the CAM, endostatin progressively elevates its expression; meanwhile, most pro-angiogenic factors show a steadily downgrading expression [36].

In addition to these endocrine and paracrine molecules and growth factors mentioned above, the extracellular matrix of the CAM may modify its composition and express fibronectin, collagen type IV, laminin and specific glycosaminoglycans. These substances can assist the angiogenesis occurring in the matrix [14].

Recently, scientists have started to reveal cellular functional mechanisms and pathways of growth factors in CAM angiogenesis, for example EGFR, Ties receptor and AKT/PKB signalling and Ras-MEK-MAPK, AKT, P38 and PKC pathways [11,37].

Besides molecular regulation on endothelial cells, effects of some molecules on pericytes and the basement membrane in the CAM have also been studied. Netrin-4 is a laminin-disrupting molecule. Through disturbing the laminin network, it disrupts the full basement membranes surrounding pericytes, which are resultantly detached from the endothelium, leading to the collapse of the capillary network. Thus, netrin-4 treatment seriously impacts CAM angiogenesis and reduces the vascular area by altering the basement membrane and pericytes [45].

## 3. CAM Assays in Preclinical Biomedical and Pharmacological Research

For over 100 years, scientists have utilised the CAM in medical studies. Rous and Murphy grafted chicken sarcoma cells onto the CAM and observed tumour growth [46]. However, it was more than half a century later when attention was drawn to vascular questions about the CAM, which is recognised as an excellent assay system for research on vascular responses [18,26,34,47]. The CAM is one of the ordinarily used in vivo models to study blood vessel development and to test and evaluate pro-angiogenic or anti-angiogenic properties of a number of substances. By utilising blood vessel features of the CAM, innovative applications have been increasingly developed, e.g., in drug screening [48] and vascular targeting therapies [49].

More recently, the CAM as a concrete experimental platform has attracted great interests from domains in bioengineering, vascular diseases, tissue transplantation, tumour and metastasis, cancer therapies, biomaterials engineering, drug development, genomics, etc. Although in ovo assays represent the main trend on a wider scale, innovative ex ovo (shell-less) CAM assays have been successfully utilised to study the angiogenic response and the initial tissue response to biomaterials engineering [50,51]. Such ex ovo modification models can keep survival rates over 80% and provide several advantages, e.g., to better visualise implanted biomaterials and growing embryos, to transplant combinatory biomaterials on the bigger area of one CAM simultaneously and to observe the vascularisation process during the whole time course of the test [51].

The CAM appears to be an excellent preclinical model for pharmacological assays due to convenient experimental manipulation, common tissue composition and economical accessibility. Meanwhile, it has been used as an intermediate step before advancing to in vivo preclinical evaluation in mammals [24]. Studies on drug pharmacokinetics and biodistribution, drug activity and toxicity are the most crucial stages in preclinical pharmacological verification, as summarised here. We also outline the best time course, advantages and limitations of CAM applications for different assays.

### 3.1. Drug Pharmacokinetics and Biodistribution

#### 3.1.1. Drug Delivery System

The CAM is regarded as an excellent platform for testing multiple drug delivery systems (DDSs), which are designed to distribute medical substances to specific sites of disease or wounds [24]. One of the major functions for a DDS is to control the releasing rate of a drug. As noted, pharmacokinetics and biodistribution in vivo are important aspects that reflect the efficacy of a DDS. A DDS can be topically applied on the CAM or injected into the amnion [14]. Topically applied drugs can enter the blood circulation after absorption by the CAM. However, recently, direct drug injection into the CAM blood vessel is being preferred [52,53], which is conformable to clinical usage.

#### 3.1.2. Conventional Analysis Approach

There are different approaches to assess pharmacokinetics and biodistribution with a CAM platform. In the traditional approach, following drug administration, blood is sampled, selected organs are extracted and then quantitative analysis of the drug can be carried out [24]. After sampling, the drug plasma level is measured by high-performance liquid chromatography (HPLC), and pharmacokinetic parameters are determined by a common algorism. After dissection and tissue sampling, the biodistribution of a drug can be evaluated by HPLC quantification of its tissue concentration in different organs [54,55].

#### 3.1.3. Advanced Approaches

The more advanced approach is either to directly observe intravascular fluorescence after injection of fluorescent materials or to utilise biosensors [24].

Regarding the fluorescent approach, fluorescent drugs can be traced by using a fluorescence microscope to measure drug penetration and distribution for pharmacokinetics biodistribution studies. The techniques for fluorescence measurement have been described previously [24,48]. After intravascular injection of a photosensitiser into the CAM, time-dependent fluorescence angiography is performed under a fluorescence microscope. The extent of fluorescence inside and outside blood vessels can be analysed by these fluorescent photographs and recorded in a semi-quantitative way. Then, by calculating the photographic contrast, the extent of the photosensitiser diffused through the CAM vasculature is determined and the profiles of photosensitiser pharmacokinetics can be outlined. 

Improved methodologies have been adopted to visualise vascular changes induced by tumours and to study tumour invasion. A metastatic human melanoma cell line (C8161) and a prostate cancer cell line (PC3) were labelled with green fluorescent protein and implanted onto the CAM. At different time points of tumour cell growth, lens culinaris agglutinin as a fluorescently tagged lectin was injected intravenously to label endothelial cells. Through fluorescent endothelial cells, microvessels were imaged and measured by confocal microscopy to evaluate angiogenesis. Furthermore, tumour cells grown on the CAM surface and invaded into the CAM endoderm could also be imaged and evaluated by confocal microscopy. Confocal z-stack imaging is a useful tool to analyse tumour invasion [56]. In another research, lens culinaris agglutinin labelled with rhodamine was injected into the blood circulation of the CAM to visualise and evaluate the microvasculature by confocal microscopy [57].

On the other hand, with biosensors, the concentration of a drug can be determined through conversion of a biological response into an electrical signal [58]. For instance, an acetaminophen sensor was topically applied and integrated into the CAM of an embryo for 7 days. Afterwards, blood levels of acetaminophen were determined with the biosensor, which reflected the changes in acetaminophen levels [59]. A biosensor was also used to measure the glucose concentration through non-invasive methods in a CAM model [60]. 

#### 3.1.4. Vessel Permeability

Vessel permeability provides an important feature to study the pharmacokinetics of drug diffusion through the CAM vasculature, while the change in vascular leakage in the CAM can be monitored at different development phases, for example during endothelial proliferation, cytodifferentiation and senescence of the chick embryo.

Vessel permeability can be measured in real time by transiting relatively small molecules into the interstitial space, which include fluorescent dextrans of about 100 kDa, macromolecular drug carriers (70 kDa) and antibodies (150 kDa), whereas larger dextrans of 2000 kDa are enclosed in the vascular space. These dextrans of different sizes provide real-time monitoring capacity to study vascular leakage in the CAM. Spatial and temporal differences in vessel permeability may also be captured by an intravital imaging approach with high resolution [15].

### 3.2. Biocompatibility

The CAM has been utilised as a model assay to study and evaluate allogenic and xenogeneic transplantations of different tissues and organs. As the earliest explorer, Murphy transplanted multiple chicken tissues onto the CAM, including the spleen, liver, kidney, bone marrow, etc., most of which survived after homologous grafting [61]. 

The CAM was also successfully transplanted with more allogenic tissues, including the liver [62], mesonephros [63], adrenal gland and cerebellum [64]. In addition, the CAM has been extensively used for xenogeneic transplantations of human bone [65], kidney [66], endometrium [67], skin [68], ovaries [69], etc. An uncompleted immune system of the chick embryo before ED 15 is the main reason for the successful results in these procedures.

In the CAM, these transplanted tissues can survive and grow by vascular anastomoses between the transplanted tissue and the original CAM and most commonly by neoangiogenesis from the CAM into implanted tissues [70]. The graft and the CAM are connected by relatively large arteries or veins; thereby, the CAM can provide grafts with nutrients and growth factors [63].

Biocompatibility is essential for tissue engineering. In recent years, the CAM has been used in innovative stem cell research. Extracellular matrices or synthetic polymers are made with desired tissue-like structures and transplanted onto the CAM without rejection, wherein cell growth, differentiation and angiogenesis can occur.

Using CAM or chick embryo to study biocompatible materials and stem cell integration has become a new future trend [15]. The angiogenic response has been tested in implanted extracellular matrices extracted from the skin, brain, oesophagus, larynx, trachea, aorta, diaphragm and cartilage, etc. These biomaterials acquire effective vascular supplies with enough oxygen and other nutrients to sustain their biocompatibility and availability [14]. 

More recently, fabricated synthetic polymers have been evaluated for angiogenesis. Biodegradable poly(lactic acid)/poly(lactic-co-glycolic acid) (PLA/PLGA) scaffolds embedded in the CAM were used to sequentially deliver VEGF, FGF-2 and PDGF with distinct kinetics in order to observe angiogenic differentiation [37]. Other biomaterials such as fibrin matrices [71], collagen matrices [72], etc., have also been used for similar purposes.

In view of the drawbacks with VEGF, such as time-consuming or complex steps and leaky or haemorrhagic vessels, new substances were explored to substitute VEGF, of which 2-deoxy-D-ribose (2dDR) and 17β-Estradiol (E2) were selected as two leads. An ex ovo CAM model was used to evaluate the angiogenic activity of the two agents, which were gradually released from tissue engineering scaffolds. Both 2dDR and E2 induced angiogenesis during 7 days and were approximately 80% as effective as VEGF. Based on dose-dependent angiogenic responses, the most effective doses of both agents were determined [57].

When non-biological materials are introduced, inflammation and ulceration could be responding problems. The trend of tissue engineering materials is to achieve biocompatibility without inflammation and to control the kinetic release of growth factors [71]. Tissue transplantation studies have also built a good basis for tumour transplantation and relevant therapies.

### 3.3. The Efficacy of Drugs

The activity or toxicity of a drug can be evaluated in the CAM and grafted tumours on the CAM [14]. To evaluate the efficacy and action mechanism of drugs in a CAM model, pro- and anti- angiogenic substances have been chosen as a pioneer area to study, including a variety of substances such as natural molecules, growth factors, anti-angiogenic molecules, antibodies, synthetic small molecules, anti-cancer agents, antibiotics, etc. [14]. 

#### 3.3.1. Pro-Angiogenic Agents

Pro-angiogenic agents can stimulate new blood vessels to grow from existing vessels, resulting in a concurrent increase in the blood vasculature. More than 140 kinds of pro-angiogenic stimulators have been tested by CAM assays [73]. To test their efficacy, pro-angiogenic agents are usually applied directly on the surface of the CAM to monitor the angiogenic response. These pro-angiogenic agents can be utilised as components of microparticles or nanoparticles, drug conjugates or tissue scaffolds [15].

There are many techniques to apply agents onto the CAM (Table 3).

Rather than placing agents on the surface of the CAM, some methods directly injecte cell suspensions or fluid substances into the allantoic vesicle so that their activity over the vascular system is uniform [86].

Growth factors of different formulations such as VEGF121, VEGF165 and FGF entrapped in fibrin matrices have been screened on a CAM platform for their efficacy in angiogenesis [71,87]. It is found that a single delivery of each of these factors resulted in angiogenic activity, but the new vasculature was leaky and haemorrhagic in the fibrin matrices. However, combined delivery of these factors or immobilising either VEGF 121 or VEGF 165 can form non-leaky vessels with a normal morphology without haemorrhages but with barrier function. These vessels look more mature than those produced as a response to any single growth factor. In addition, VEGF alone can initiate the formation of structurally intact vessels, provided it is released slowly in a low sustained dose [87].

#### 3.3.2. Anti-Angiogenic Agents

As an in vivo model, the CAM has also been utilised to study the efficacy of anti-angiogenic modulating agents. The efficiency of anti-angiogenic drugs or formulations was evaluated by determining the extent of vascular occurrence in the CAM [88], the reduction of blood vessels [24] and the presence of an avascular or a hypovascular zone at the drug application site [89]. 

Around 360 anti-angiogenic agents have been tested in CAM assays [73]. They are classified into two groups (Table 4): (a) direct inhibitors, which affect the survival or functions of endothelial cells, and (b) indirect inhibitors, which block the activity of pro-angiogenic molecules [89]. These are semi-synthetic or synthetic substances, biological antagonists or endogenous factors which inhibit the immature neovasculature or the angiogenic cascade [89].

Avastin (bevacizumab) is a typical example of anti-angiogenic agents in clinical application. It is an anti-VEGF-neutralising monoclonal antibody and an anti-angiogenic agent which is first utilised in clinical anti-cancer therapy [52,89]. 

Avastin has been extensively studied for its efficacy by using CAM assays [52,90]. It was applied onto the CAM at ED 7, and fluorescence angiography was used to observe vascular changes through ED 9. Comparing images at EDs 7 and 9 showed that Avastin strongly inhibited angiogenesis in the CAM [52] and caused an increment of over 32% of the mean avascular surface as compared to the control group. Avastin also caused a significant reduction in the capillary density at the tested CAM site [52].

The yolk sac membrane (YSM) was also used to substitute the CAM to test Avastin. After Avastin treatment, the number of quaternary blood vessels in both the YSM and the CAM significantly decreased, which validated pilot screening for anti-angiogenic agents [88].

Sunitinib is an inhibitor for multiple receptor tyrosine kinases and can work excellently to inhibit angiogenesis. It can be used as a negative control for angiogenic responses compared with tested agents and a positive control (VEGF) in the CAM [57].

Another good example is paclitaxel, an anti-cancer drug with anti-angiogenic property. The efficacy of several formulations of paclitaxel was analysed on a CAM model [24]. Paclitaxel was incorporated into microspheres made from either poly(ɛ-caprolactone) (PCL) or Poly lactic acid-ethylene-vinyl acetate (PLA-EVA) copolymer. By using PLA-EVA microspheres, the paclitaxel dose was about eight times smaller than the dose used for PCL microspheres in order to induce the same level of vascular occlusion. These results demonstrated that the CAM model can compare the performance of drugs with different carriers. Some formulations were tested to adjust the release rate of paclitaxel in a CAM assay where methoxypoly ethyleneglycol (MePEG), PCL, lipospheres and gelatine were applied to validate its pharmacokinetics [24].

Other innovative anti-angiogenic agents have also been explored on the CAM for photodynamic therapy (PDT) and radiotherapy. PDT is to combine photosensitisers (anti-angiogenic compounds) with various activating light sources to suppress vascular growth, whereas radiotherapy increases the effectiveness of anti-angiogenic treatment when X-rays are combined with radio-sensitisers [91,92].

#### 3.3.3. Wound Healing

The US Food and Drug Administration (FDA) used the CAM assay to preclinically evaluate drugs to be approved for treating burn wounds and chronic cutaneous ulcers [93]. The CAM was used to study wound healing [94]. The CAM could constantly reproduce all stages in human wound healing, including inflammation, re-epithelisation, angiogenesis, fibronectin deposition and scar formation. During wound healing on the CAM, chorionic epithelium hyperplasia and macrophage inflammatory infiltration can be observed with tripled micro-vessels and fibroblasts in the wound area than in the adjacent control area. 

A CAM assay may also test the mechanism of action of specific substances during wound healing. Taking FGF-2 as an example, blocking antibodies were used to inhibit FGF-2 after CAM wounding, which inhibited the development of micro-vessels and fibroblast density. Thus, wound-healing delay was observed. Conversely, when FGF-2 was added into the wounds, the repair was accelerated by 24 h compared to control wounds [95].

Another substance, activated protein C (APC), which is a serine protease anti-coagulate and a potent anti-inflammatory mediator, was tested for wound healing [96]. Via a complex mechanism, APC may help cutaneous wound healing by angiogenesis stimulation, re-epithelialisation promotion and inflammation inhibition. This CAM assay suggests that APC can be an interesting therapeutic to accelerate chronic wound healing.

In silico models have become innovative tools to mimic and understand cell behaviour before real animal assays start. Concerning wound healing, an in silico numerical model accurately simulated the sprouting angiogenesis process, which was regulated by the chemoattractant effect of VEGF [97]. It was the first time to simulate the capillary network presented in realistic CAM images. The basic methodology was to integrate the radial point interpolation method (RPIM) with the characteristics of growth factor initiation, endothelial cell movement and branching pattern. Through a comparison and validation between the in silico model and the in vivo CAM, the parameters (total branching number, total vessel length and average branching angle) were closely simulated. The capillary volume fraction was also compared. The in silico model made sprouting angiogenesis more predictable and the combination with the in vivo assay more valuable [97].

#### 3.3.4. Tumour Growth and Metastasis

A. Tumour angiogenesis and growth

The CAM is an excellent host to study cancer biology and tumour graft growth, because the immune system has not become competent and rejection has not been established until ED 18 [73].

In 1913, Murphy first explored tumour-induced angiogenesis with the CAM [98]. Afterwards, others attempted implanting tumour cell lines or tissues from mice, chickens and humans on the surface of the CAM in their studies [73,99,100,101]. Thereby, characteristics of tumours could be observed, such as tissue development, angiogenesis, invasion, extravasation and metastasis [15,101]. This makes it feasible to compare and evaluate tumours’ growth and histological features as well as their viability after being re-transplanted in the original host and the effects on chick embryos [100].

The CAM tumour assay is much faster than a tumour assay in mammalian models, which often take 3–6 weeks [14]. After tumour cell inoculation onto the CAM, the grafts remain avascular for a few days, after which they are ready for rapid growth once penetrated by new blood vessels. Normally, it takes 2–5 days for tumour xenografts to become visible with their blood supply originating from the CAM. 

A detailed process of tumour tissue transplantation has been described previously [102]. Tumours grafted on the CAM do not surpass a mean diameter of 0.93 ± 0.29 mm during the prevascular phase (approximate 72 h). After entering the vascularisation phase, it takes 24 h for tumours to start growing quickly and reach a mean diameter of 8.0 ± 2.5 mm by 7 days. Interestingly, when 1–4-mm-size tumour grafts are implanted on the CAM at ED 9, they undergo a prevascular phase for 72 h and shrink rapidly due to necrosis and autolysis. However, when vascularisation starts, rapid growth resumes.

Through a morphological study, the blood vessels in grafted tumours and CAM host blood vessels were distinguished [70]. The pre-existing tumour native blood vessels in the graft disintegrate in 24 h after implantation, and vascularisation reoccurs when CAM-derived vessels penetrate into the graft. This mechanism is completely different from the one in the grafts of chick tissue or adult tissue.

B. Tumour metastasis

The CAM can practically host the growth of inoculated xenogeneic tumour cells and tissues, which efficiently helps to simulate and analyse metastases of human tumours [99].

Cancer metastasis is initiated by a change in cell–cell adhesive interactions [73]. During this process, tumour cells first dissociate from the primary lesion, undergo local invasion and migrate into the interstitial matrix. For haematogenous metastasis, tumour cells enter the host circulation through intravasation, get stagnated in the microvasculature and leave the blood circulation by extravasation. Then, the tumour cells may start local invasion again to form secondary metastatic foci. Finally, these small foci may initiate an angiogenic response for sustaining tumour growth. 

Undergoing such metastasis, tumour cells can colonise in the CAM and in the internal organs of the embryo, such as the brain, lungs and liver [103]. These metastatic cancer cells simulate stem cells because of their ability to self-regenerate and to derive diverse next generations [73]. Such a small group of cancer cells is named cancer stem cells with stem-like properties. Organotropic breast cancer cell lines were studied for such active properties [104]. The evaluation was undertaken by a limiting dilution assay in the CAM, following in silico bioinformatics analysis, to validate the stem-cell-like prevalence of different breast cancer cell lines as an updated approach to determine the stem cell properties of tumour cells.

When studying metastasis either by a spontaneous model or by an experimental model, as described below, the chick embryo only takes 7–8 days, considerably shorter than 4–10 weeks with most typical mice models [73].

*Spontaneous metastasis model*

Spontaneous metastasis is the process by which grafted tissue or inoculated tumour cells intravasate into the vasculature of the CAM, which shows the metastatic potential of a specific cell line or primary tumour fragment responsible for tumour progression [15,73].

Histological analysis of the CAM may reveal that tumour cells invade the chorionic epithelium from the surface and the blood vessels through the intermediate mesenchyme [73,103,105,106]. 

Tumour cells invade the vascularised mesenchyme underneath the chorionic epithelium. By attaching to arterioles, cancer cells migrate to the dense bed of blood vessels to realise intravasation. In vivo microscopy can monitor morphological changes in cancer cells present in the CAM microcirculation, which mostly survive without significant cell damage. On the CAM, tumour cells can be found in locations away from the inoculation site and in internal organs within a few days after inoculation [11,14].

*Experimental metastasis model*

Experimental metastasis refers to injecting cancer cells into the blood circulation to form metastatic tumours in distant organs. Experimental metastasis surpasses dissemination steps of spontaneous metastasis [107]. Experimental metastasis studies on the CAM may provide comprehensive information about critical metastatic steps, including (i) cancer cell survival in the blood circulation and transportation to target organs, (ii) cancer cell immobilisation in the microcirculation, (iii) cancer cell migration through the vessel wall into the interstitial space (extravasation) and (iv) cancer cell proliferation in target organs [73].

By comparing spontaneous and experimental metastases, different functions of non-metastatic and metastatic cells can be determined at the steps of the dissemination cascade [99]. The CAM is an attractive model to visualise the behaviour of grafted tumour cells with microscopy in the settings of both spontaneous and experimental metastases.

Another mechanism of cell dissemination is angiotropism, in which tumour cells preferentially localise in the existent blood vasculature, rather than randomly migrating within the CAM mesoderm [85].

C. Drug assays

Anti-cancer drug therapies can be evaluated when tumours are grafted on the CAM [14]. The CAM has been used as a test platform for anti-angiogenic drugs to study the inhibition of angiogenesis of tumour growth [11], chemosensitivity towards tumour invasion and metastasis [101,108], inhibitory effects of chemotherapeutic drugs on metastatic foci [109] and experimental radiation oncology research [110]. Common CAM-based drug assays are listed in Table 5.

Since the CAM is an isolated subject without external excretion, the metabolic half-life of many molecules, such as small peptides, tends to be much longer in the chick embryo compared to other animal models. This makes it feasible to experimentally evaluate potential anti-metastatic compounds available only in small quantities [14,73].

### 3.4. Screening of Drug Toxicity

#### 3.4.1. Irritation

The CAM assay has been accepted for testing the drug irritation potential on human skin. Traditional animal sensitisation approaches can be altered by the promising chimeric skin–CAM system. The grafted skin remains viable as long as the survival of the chick embryo with a constant blood supply [68].

The CAM is accepted to substitute the Draize test, which tests the potential irritation by chemicals on rabbit eyes [14]. The Draize eye irritancy test placing substances into the eyes of rabbits can now be replaced by the CAM to determine the irritation potential of liquid, flake and powdered cosmetics [117]. 

In a comprehensive safety report, 32 chemicals and 187 cosmetic products were evaluated by using CAM irritation tests. A good concordance was observed between CAM and in vivo tests for all substances. In addition, the CAM can be a screening tool for cleansing foam, hair-dye, hair-curling and shampoo formulations [118]. 

In addition, the CAM is an alternative platform for irritation testing of ocular tissues such as the cornea and conjunctiva, because they share similar responsive inflammatory reactions to irritant substances. By using the CAM, naltrexone, as a therapeutic agent for diabetic keratopathy, has been tested for its irritant effects on blood vessels [119]. A new molecule LQFM048, as a photoprotective agent, was also tested on the CAM with a purpose to investigate its eye irritation potential. Measured parameters include haemorrhage, coagulation and vascular lysis, resulting in no irritation on the CAM test [120].

#### 3.4.2. Toxicity

The adverse effects on various organs, damage to the vasculature and even embryo death can be evaluated as indexes for the toxicities of tested drugs or carriers on the CAM [14]. Here are a few examples.

The influence and toxicity of cigarette smoke and nicotine were investigated in an ex ovo chick embryo culture [121]. Shell-less chick embryo cultures were used to assess the effects of acute glucose toxicity [122]. The toxicity of certain herbal medicines such as Angelica sinensis injection (ASI) and Astragalus membranaceus injection (AMI) was explored on a CAM platform [123]. Control, ASI, AMI and different ratios of ASI and AMI were applied topically on the CAM via the drug carrier. After treatment, the number of living chick embryos was counted. Results showed that ASI and AMI did not inhibit the survival of chick embryos, and it was concluded that ASI and AMI could be further investigated for clinical applications in angiogenesis modulation.

As an advanced anti-cancer therapy, the combination of a drug and a carrier can be assessed. Biodegradable periodic mesoporous organosilica (PMO) nanoparticles were developed, and doxorubicin (anti-cancer drug) was loaded inside. Human ovarian cancer cells were planted on the CAM to induce rapid tumour formation. The efficacy and toxicity of the drug were evaluated after nanoparticles were injected intravenously into the embryo. The tumour underwent elimination, and no significant damage to various organs (heart, liver, spleen, kidney, lung and intestine) occurred in the embryo. Toxic effects of the drug-loaded nanoparticles were obviously missing. In contrast, when doxorubicin without nanoparticles was injected directly, widespread organ damage was observed, even when a low concentration was administered [53]. These results sugges that the CAM is a valuable tumour platform to screen the toxicity and side effects of novel drugs and therapies.

#### 3.4.3. Anti-Vascular Selectivity

The vascular system is vital to supply nutrients to normal tissues and particularly tumours, so vascular-targeting therapy is a main branch in anti-cancer therapies. The toxicity and side effects of vascular-targeting drugs and substances must be first screened at preclinical research levels.

*Vascular-targeting strategies*

Vascular-targeting therapies (VTTs) include two different approaches: the anti-angiogenic approach and the vascular-disrupting approach [116]. Accordingly, there are two distinct categories of agents: anti-angiogenic agents (AAs) and vascular-disrupting agents (VDAs). AAs suppress the growth of the new tumour vasculature through the inhibition of vascular endothelial growth factor (VEGF) and other pro-angiogenic molecules [3,124], whereas VDAs act on endothelial cells and pericytes of the existent tumoral vasculature and consequently vessel occlusion, shutdown of the circulation and pervasive necrosis are induced inside the tumour [3,125].

*Vascular-disrupting agents*

VDAs are a type of novel vascular-targeting agents used to treat solid cancers. VDAs can selectively act on the tumour vasculature by several pathways by which blood flow inhibition and extensive secondary necrosis occur within tumours, while having little effect on non-malignant tissues (which remain relatively intact). Tumour blood vessels are abnormal in structure and function, which are fundamentally different from the vascular networks of normal tissues, and thus selectively targeted by VDAs [3,125,126].

*Classification and mechanisms of VDAs*

Currently, two types of VDAs exist in preclinical and clinical R&D, small molecules and ligand-directed VDAs. Small-molecule VDAs are in the advanced stage of clinical trials, which can be further divided into two categories: tubulin-binding agents and flavonoids [125] with distinct mechanisms of action.

A. Tubulin-binding agents

**Figure 8:**
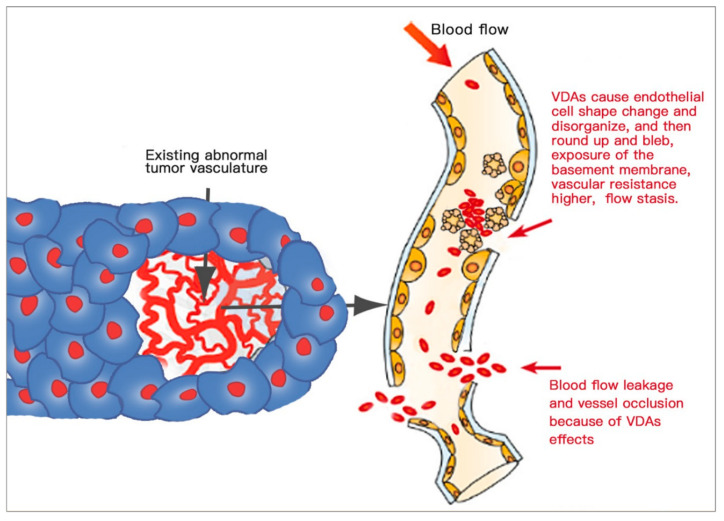
Schematic mechanisms of tumour vascular-targeting tubulin-binding agents.

Tubulin-binding agents act directly at the endothelial cytoskeleton, bind to the colchicine-binding site of the β-subunit of endothelial tubulin, initiate microtubule depolymerisation and actin/tubulin disorganisation, disrupt endothelial conjunctions and cause cytoskeleton deformation and dysfunction. As a result (Figure 8), endothelial cells round up and bleb on the surface, leading to increased vascular resistance, blood flow stasis, blood cell stacking, leaky vasculature and intra-tumoral vessel occlusion, followed by coagulation cascade, thrombus formation, cell–cell disconnection and exposure of the already abnormal basement membrane. Ultimately, massive ischaemic and haemorrhagic tumour necrosis occurs [3,126,127]. 

It must be emphasised that (1) such VDA-induced cytoskeletal disruption occurs selectively in immature endothelial cells with a lack of contact with smooth muscle and pericytes, instead of vessels in normal tissues, and (2) such VDA-induced tumour necrosis is always incomplete, leaving viable cancer cells behind for tumour recurrence [3], which presents a bottleneck problem with VDAs and calls for a smart solution [49]. 

Representative tubulin-binding agents are stilbenes of the combretastatin family and heterocyclic compounds, including combretastatin A-4 phosphate (CA4P), a combretastatin A-1 derivative OXi4503 and a serine-linked amino derivative AVE8062 [127], as listed in Table 6.

B. Flavonoids

ASA404 or DMXAA (an analogue of flavone acetic acid) is an example of this class (Table 6). Flavonoids have a mechanism which is tubulin independent but induces both direct and indirect anti-vascular action. The direct action leads to rapid apoptosis of endothelial cells, probably due to the induction of tumour necrosis factor-α and the recruitment of activated neutrophils, whereas the indirect action stimulates the production of cytokines, for example tumour necrosis factor-α and other cytokines and chemokines, resulting in ischaemic tumour necrosis [3,126].

*VDAs under research and clinical development*

Several small-molecule VDAs have been assessed for cancer treatment. However, the development many VDAs was terminated because of their unsatisfactory efficacy or their toxicity and side effects (Table 6).

Combretastatin A4-phosphate (CA4P) is a most extensively studied VDA in clinical trials for various tumour types, alone or in combination therapy. Thus, CA4P is chosen to demonstrate the features, efficacy and safety of VDA treatment.

Many trails on a single use of CA4P in human patients have been performed. CA4P causes an acute and serious shutdown of blood vessels and nearly overall stopping of blood flow in the tumour and ultimately leads to selective tumour necrosis. However, such impacts are not present in normal tissues. Meanwhile, CA4P is safe and well tolerated and lacks haematologic toxicity, which is verified by studies under different dosing schedules (weekly, 3-weekly, and daily for 5 days every 3 weeks). A maximum-tolerated dose (MTD) of 50–60mg/m^2^ was set with consistent anti-vascular effects [125]. Through several preclinical studies on dose–time correlations of CA4P treatment in tumours and normal tissues, an estimated dose of 10 mg/kg of CA4P was set as the MTD for rats, equivalent to that for humans [138].

It must be noted that CA4P, list other VDAs, can cause considerable tumour necrosis, but tumour regrowth is attributed to peripheral sparing cancer cells (the viable rim) which are supplied with nutrients and oxygen by the surrounding normal vasculature. Rapid re-growth of tumours can ensue when a single VDA is administered [125,138]. It is a common drawback of VDAs, which needs to be tackled [49]. These facts indicate a direction: VDA combination therapies, for example CA4P plus chemotherapy, CA4P plus AAs, CA4P plus AAs and chemotherapy, CA4P plus radiology, etc.

Combined with chemotherapy, CA4P showed promising results in clinical trials [3]. A safety and efficacy evaluation was carried out for CA4P with paclitaxel/carboplatin in anaplastic thyroid carcinoma. The results showed that one-year survival for chemotherapy/CA4P versus chemotherapy alone was 26% versus 9%, respectively. In addition, no significant cardiovascular side effects arose and the therapy was well tolerated. Thus, CA4P with carboplatin/paclitaxel can be a prospective therapy for anaplastic thyroid carcinoma [139].

Patients with platinum-resistant ovarian cancer underwent a phase II trial with CA4P in combination with carboplatin and paclitaxel. CA4P with paclitaxel and carboplatin caused a higher response rate in patients than sole chemotherapy without CA4P and was well tolerated [140].

The VDA–AA combination could be an effective treatment without chemotherapy. Human clear cell renal carcinoma tumours were tested. With the VDA–AA combination, there was a significant tumour response than with single-agent treatments. CA4P plus bevacizumab (Avastin) realised a tumour growth delay of 13 days [141].

The safety of CA4P in combination with carboplatin, paclitaxel and bevacizumab (an AA) was studied in patients with advanced nonsquamous, non-small-cell lung cancer (NSCLC). Overall and progression-free survival rates were comparable in groups, and CA4P plus carboplatin, paclitaxel and bevacizumab appears a tolerable therapy with acceptable toxicity [142].

There is an emerging interest in combining VDA therapy with AAs and chemotherapy together, especially for patients with poor prognosis. Treatment for platinum-resistant ovarian cancer was proposed [143]. Mateon Therapeutics Ltd. also submitted clinical trials to the FDA (clinicalTrials.gov identifier NCT02641639). However, all these clinical trials so far have failed to be approved by the FDA for commercialisation due to insufficient therapeutic efficacy.

More recently, by combining a VDA with a radioactive necrosis-avid compound, a dual-targeting broad-spectrum anti-cancer therapy, OncoCiDia, was introduced, which is supposed to solve the bottleneck problem of incomplete tumour destruction with all VDAs [5].

However, there is a pending critical uncertainty from scientific and clinical viewpoints: tumour specificity [125]. Do VDAs selectively act on the tumour-related endothelium, or is there a more general effect on normal blood vessels with a risk of subsequent ischaemic complications and other safety risks? This uncertainty could be tackled by utilising a CAM research platform.

*VDAs assays on the CAM*

So far, only a few researchers have explored the CAM model to evaluate vascular responses of VDAs (Table 7).

These studies have just been reported with preliminary evaluation of the vascular responses towards VDAs. Testing methods are simple with VDAs applied topically on the surface of the CAM to observe visual changes in vessels and their branches and to count the number of blood vessel branches by photography [130] and even manual counting [144]. There have not been any more precise quantitative analyses of the vascular response to VDAs, with a lack of analyses on efficacy and safety.

### 3.5. Time Course of CAM Applications for Different Assays

To achieve the best results of preclinical assays, a specific time course (ED period) of the chick embryo is important to be defined for different CAM applications. 

#### 3.5.1. Drug Tests

Commonly, a drug can be applied on the surface of the CAM or injected into the amnion and the allantois at EDs 7 and 8 [24] in order to examine the drug’s efficacy and toxicity. However, drug topical application appears more flexible in terms of ED periods. In one study, drugs for primary pancreatic cancer cells were tested. Tumours grown from pancreatic cancer cells were treated with gemcitabine or crizotinib or their combination, topically applied daily from ED 10 until ED 18 on the CAM [146].

Other researchers prefer ED 9 as the optimal time to inject drugs intravenously into the CAM to test drug formulations, since major blood vessels are mature at this time [147]. We believe this period can be expanded until ED 10. 

#### 3.5.2. Vascular Assays

The period of ED 11 until ED 13 has been opted for assays targeting the existent vasculature [147,148]. This was also concluded and recommended from our own practical experiences. After ED 10, major vessels mature and become stronger, so it is more convenient for vascular interventions and imaging monitoring. However, beyond ED 13, it becomes difficult to illuminate through the entire egg to find the exact position of vessels.

For neovascular assays, the chosen time window is from ED 5 to ED 10, although it was found that angiogenesis decreases as early as ED 5 by hyperglycaemia treatment [149]. 

Renal tumour fragments and endostatin were placed on the CAM on ED 7, and their interactions were examined on the vascular network [150], while another study applied gelatin sponges soaked with a blood vessel stimulator or inhibitor on the developing CAM at ED 8 [89].

The quantity of intussusceptive pillars in the CAM peaked between ED 8 and 10 [40,151]. On ED 8, the capillary plexus appeared much denser, and numerous intercapillary tissue islands started to be detected. Therefore, EDs 8–10 are more appropriate for neo-microvascular assays.

#### 3.5.3. Tumour Implantation

When reviewing the CAM model for tumour biology, a strong angiogenic response occurs when tumour tissues are implanted on ED 8 to ED 10 onto the CAM, but there is no such response on ED 11 to ED 12 [152]. This can be explained by the high mitotic rate of CAM endothelia until ED 10, followed by a decrease later. Osteosarcoma cells were implanted onto the CAM at ED 9 [153], and urological cancers cells were implanted onto the CAM on ED 10 [154]. Although HuH7 liver cancer cells were successfully implanted on the CAM since ED 7 [155], we still recommend EDs 8–10 as a good time course for tumour implantation. 

#### 3.5.4. Cell Growth Factors and Antibody Inhibitors 

Similar to the vigorous period for CAM angiogenesis during EDs 7–12, this time window is also optimal for the investigation of the effects of cell growth factors and antibody-mediated inhibitors [37]. Vascular endothelial growth factor (VEGF), fibroblast growth factor (FGF) and platelet-derived growth factor (PDGF) are among classic cell growth factors.

Recently, stem cells and their derivatives were tested on the CAM for new exploration of tissue engineering. Although ED 5 and ED 11 have been tested, the CAM at EDs 8–12 is considered to yield the most significant impacts of stem cells on angiogenesis [156], which is identical to the conclusion of Bai et al. [37]. An antibody, anti-FGF2 (fibroblast growth factor inhibitor), was tested on the CAM at ED 8 to treat myeloma plasma cells and cancer metastasis [152]. A neutralising antibody (Protein A) was validated for its effects on OV-90 ovarian cancer cells implanted onto the CAM at ED 11 [116].

### 3.6. Advantages and Drawbacks

The CAM as a novel test platform possesses both advantageous features and certain limitations, as summarised in Table 8. 

## 4. Methods to Image and Evaluate the Changes in the Vasculature of the CAM

Advanced imaging techniques have enabled us to inspect and quantify the number, spacing and growth of blood vessels on the CAM, analyse their structural and functional normalities or abnormalities, measure blood flow and vascularity and facilitate the assessment of vascular-targeting drugs in laboratory or preclinical assays [157].

The CAM is one of the most broadly used assays for vascular-related research [1]. Therefore, various imaging and evaluating techniques have been developed for determining the angiogenesis and vascular changes induced by different factors or substances applied on the CAM.

The most conventional methods are destructive: the vasculature of the excised CAM is observed through a microscope or camera; another approach is morphometric analysis of the blood vessel network based on stereology [158]. Under such conditions, the CAM is not intact, not in vivo, and the chick embryo is not vital.

Non-destructive techniques have been developed to provide versatile images of the CAM vasculature. These can be divided into X-ray, magnetic resonance, gamma-rays and acoustic and optical techniques, supplemented most recently by artificial intelligence (AI). Those modalities can, respectively, measure structural changes in the vascular system, the number and diameter of blood vessels and certain functional parameters, for example blood flow, dynamics and blood oxygen levels [44,157].

Innovative methods for imaging the vasculature can visualise blood vessels in tumours grafted on the CAM in order to assess the efficacy of vascular-targeting drugs for treatment of cancer or other chronic diseases.

### 4.1. Light Microscopic Methods

Microscopies are traditional and powerful methods to visualise vascularisation and vascular changes. Furthermore, new contrast or imaging agents selectively label certain vessels, which enable one to visualise selectively specific vasculature at the microscopic level.

By microscopic methods, the simplest evaluation is to observe the presence or absence of blood vessels and their changes with parameters such as the number or density of blood vessels; vessel dimensions, including length, diameter and branch points; and the total area of the CAM [44,73,157]. Vascular changes can be determined by using stereological principles and morphometric analysis. Qualitative, semi-quantitative and quantitative skills are available, among which automated image analysis and statistical post-processing methods are valuable tools incorporating appropriate computer software [44,157,159]. A ×10 magnification stereomicroscope was utilised to analyse the vascular convergence towards the graft, distribution, branching and density of CAM vessels at different time points. A ×250 magnification microscope was used to quantitatively evaluate microvessel density, which is expressed as the percentage of intersectional points occupied by microvessels [159].

Different tumour cells were inoculated into the CAM. Macrovessel images next to the graft were taken by a handy digital camera at ED s10, 12 and 14, and the images were processed to quantify angiogenesis. In parallel, microvessels were evaluated by confocal microscopy, which focused on the capillary plexus adjacent to the surface of the CAM [56].

A fluorescent microscope was also used to visualise expansion of the metastasis in the CAM. The human epidermoid carcinoma cell line was injected intravenously, and a fluorescent stereomicroscope imaged a section of the CAM at different intervals over days. Contrasting the dark-green background from the eggshell, the tumour cells were visible as bright-green spots and the vessels were visible as black lines [160].

One set of intravital microscopy was designed to image blood vessels which grew or regressed in the CAM and to inspect neovascularisation in tumour grafts in the ex ovo chick embryo [161]. It could catch real-time images for over 72 h and quantify the angiogenesis progress, without affecting the host and tumour systems. This designed unit was integrated with an upright epifluorescence or spinning disk confocal microscope. Fluorescent nanoparticles or dyes were used to enable visualisation of blood vessels. Such intravital microscopy can collect 3D spatial and temporal data. Rapid successive fluorescence images can be acquired in a single plane to visualise the dynamics of blood flow. The detailed structure at specific time points can be analysed by a 3D image stack at high resolution. Vasculature changes over time can be figured out by acquiring 3D stacks. Meanwhile, intravital microscopic imaging could display several unusual features of tumour vessels in preclinical applications [157].

The microscopic methods provide the highest resolution; confocal microscopy has about 100 nm resolution, and scanning electron microscopy (SEM) yields a few nanometres, which is only restricted for ex vivo studies [157,161].

### 4.2. X-ray Methods

With the aid of a contrast medium, computed tomography (CT) is conventionally used to document vascular characteristics such as blood flow, blood volume, mean transit time of flow and microvascular permeability. Normally, a greyscale image is obtained by means of contrast agent injection and the X-ray attenuation coefficients of the tissues. By computer processing of a series of 2D sections and tomographic reconstruction, one 3D image of the inside of the subject is generated [162,163]. Moreover, dual-energy CT, also named spectral CT, is a novel technique which uses two independent X-ray photon energy spectra. Materials with different attenuation properties can be interrogated at different energies [164]. It could be applied for studies on a CAM platform.

In one study, 3D micro-CT was used to explore three topics on embryonic viability and morphogenesis: (1) impact of the micro-CT X-ray radiation dose, (2) impact of the CT contrast agent type and microinjected dose and 3) CT contrast media biodistribution and persistence within the embryo. It was demonstrated that micro-CT is feasible for analysing 3D volumetric quantitative imaging of live embryo morphogenesis and that the efficacy and safety of contrast-enhanced micro-CT appears acceptable for live embryos [165].

Digital substraction angiography (DSA) is another X-ray-based imaging acquisition technique to visualise the vascular system of the CAM in the developing chick embryo [166]. DSA is a dynamic technique which can effectively demonstrate blood flow patterns at various phases of circulations in the CAM and chick embryo: from the CAM vein to the heart and then to arteries of the embryo and CAM membrane arteries, etc. It is able to determine vessels with a diameter of 100 µm and study morphological changes in the vasculature in the embryo system. A modern DSA system utilises low-noise video and digital electronic equipment with high-speed image processors to assess vascular effects of various therapies. DSA is also used to evaluate the effect of ionising radiation on the angiogenesis process in the CAM. Computerised analysis of angiographic images showed that the anti-angiogenic effect of irradiation during various phases of CAM development is insignificant during the studied time window [167]. Furthermore, the anti-angiogenetic effect of ionising radiation on the angiogenesis of a transplanted tumour on the CAM was evaluated and quantified by using DSA [168]. Irradiated areas of micro- and macro-vessels (smaller or greater than 200 µm) presented a damageable effect reflecting statistical reduction in both length and total vessel area.

### 4.3. Magnetic Resonance Methods

Regarding magnetic resonance imaging (MRI), the object is placed in a strong electromagnet, where the hydrogen atoms align parallel to the magnetic field. A short powerful radio signal is released passing through the object, which is perpendicular to the electromagnetic field. The hydrogen atoms which have the same frequency as the radio signal get excited and start to resonate with the exciting wave. When the radio signal is switched off, the hydrogen atoms return to their original energy state after a certain period of time. Meanwhile, the absorbed excitation energy is then released in the modality as radio signals, which are detected to acquire an MRI picture at this level or slice. The releasing time depends on the characteristics of the tissue and the number of atoms and is measured and configured into 2D and 3D images [163]. The spatial resolution of clinical MRI is 100 to 500 μm. Micro-MRI has better spatial resolution, up to 10 μm, in preclinical modalities, with poorer temporal resolution [157]. MRI can measure the blood volume and blood vessel permeability through contrast-agent-enhanced dynamic imaging [163].

Combining specific and sensitive probes, high-resolution MRI is regarded as a promising technique for molecular imaging [169]. Three-dimensional magnetic resonance microscopy (MRM) has been successfully developed to image perfusion-fixed hearts of chick embryos. The major heart chamber and even blood vessels of smaller diameters can be visualised after the perfusion of the embryonic cardiovascular system. The technique enables volumetric analysis of chick heart morphology at different stages. 

MRI was utilised to study tissue bioengineering by monitoring bone implants on the CAM. The bone formation process was assessed in polymeric scaffolds in vitro, which had been implanted onto the CAM. Furthermore, to improve the in vivo assessment, a novel MRI contrast agent was developed to facilitate detection of mineral deposits within engineered bone with improved specificity [170].

Another innovative application of MRI is to monitor and quantify the perfusion capacity of 3D biomaterials in a model of a scaffold placed on the CAM in vivo and non-invasively [171]. The perfusion capacity was assessed by comparing the longitudinal relaxation rate before and after injecting a paramagnetic MRI contrast agent. The relaxation rate changes have a strong positive correlation with vessel density, which was histologically proven, whereas vessel density correlates with perfusion capacities. The highest relaxation rate changes were found at the interface where the scaffold was attached to the CAM, and the surface of the scaffold showed the lowest relaxation rate changes. In addition, a large difference of perfusion capacities can be observed among different biomaterials, suggesting that MRI is sensitive to reveal such differences [152]. Therefore, by means of MRI in vivo, the CAM can be a good platform to test a large variety of bioengineered materials.

**Figure 9:**
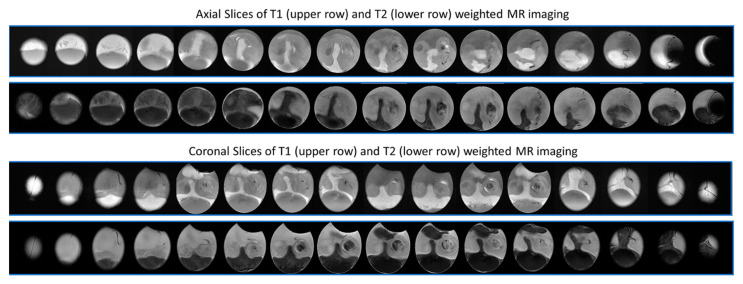
Using a clinical 3.0T Siemens magnetic resonance imaging (MRI) scanner, a chick embryo on ED 12 can be imaged entirely at both axial and coronal slices of 3 mm thickness with both T1 and T2 weighted sequences, reflecting different physiochemical properties of the tissue components.

Unlike X-ray- and gamma-ray-based imaging modalities, which emit ionising radiation harmful to organisms, including animals and humans, MRI is virtually harmless to life and therefore can be applied to monitor the growth of chick embryos longitudinally or on any ED (Figure 9).

### 4.4. Gamma-Ray Methods

Positron emission tomography (PET) is a new approach utilising positron and gamma rays. A positron-emitting radiotracer is intravenously administered, and its distribution is monitored during a suitable uptake period. While decaying, the radionuclide emits a positron, which interacts with an electron in the environment, inducing the emission of two gamma rays in opposite directions. These emissions can be detected by two detectors on opposite sides of the object [163].

For a normal tissue study, PET can measure the blood volume by the signal but cannot actually visualise vascular structures [157]. For cancer studies, tumour functional characteristics such as blood flow or volume and glucose metabolism can be accurately quantified by the application of radiotracers, e.g., ^15^H_2_O, ^11^CO and ^18^FDG [163].

Tumour glucose metabolism and protein synthesis were successfully imaged by PET in a U87 glioblastoma model on the CAM. The injection of ^18^FDG into blood vessels of the CAM enabled dynamic imaging of glucose metabolism, and the uptake of ^18^FDG over time in individual tumours was verified [172]. The accuracy of tumour volume measurements can be improved by CT imaging. Therefore, a combination of PET and X-ray CT becomes a standard approach to ensure and elevate the accuracy of imaging, which yields functional data with anatomic information. In addition, the novel application of PET/CT was tested on the CAM to screen novel PET tracers [172]. A CAM tumour model is particularly valuable for rapid and high-throughput screening of novel radiotracers, as well as for optimisation of PET/CT imaging protocols [153].

### 4.5. Acoustic Methods

Ultrasonography refers an ordinary and widely available imaging modality in both clinical and preclinical research. It is suitable to visualise tumour growth and vascularisation in the CAM assay. By using a commercial ultrasonographic scanner, tumour growth and angiogenesis were successfully monitored, and results of ultrasound images were significantly correlated with those of histological analysis from the excised tumour [155].

Photoacoustics is based on the generation of sound waves by laser radiation. A short laser pulse heats absorbers inside the tissue, with the temperature raised by generated energy. Due to up-and-down changes in the temperature, the absorber expands and subsequently contracts, inducing a pressure wave or an acoustic wave. These time-trace waves disperse through the tissue and can be seized by the detector at the tissue surface. The dispersion time determines the photoacoustic position [173]. The strength and the profile of the captured signals carry the overall spatial information about the distribution of laser absorption in the tissue [174].

Blood (haemoglobin), a typical absorber of ultrasound, can position and monitor blood flow and concentration in vessels, tissues and tumours. A photoacoustic system was used to visualise single blood vessels in the CAM at ED 9, which revealed that the majority of vessels were sized from 200 to 500 µm in diameter, with other smaller vessels ranging from 125 to 200 µm. Meanwhile, through 3D rendering of the reconstructed dataset, the blood vessels’ structures of 3.5 mm depth became clearly visible [173]. In most cases, photoacoustic signals were acquired at flat and reflective surfaces. However, in real biological applications, the signals always interfere with the roughness of sample surfaces. Considering this fact, a fibre-based noncontact photoacoustic tomography system was designed to overcome the disadvantage. With a fibre-optic heterodyne interferometer as a signal detector, the system measured the surface displacement of a sample. In fact, the system could successfully measure the photoacoustic signals from the surface without any physical contacting with the sample [175].

A similar principle called photothermal radiometry, which uses thermal waves to image discrete blood vessels of the CAM in vivo, was applied. However, the authors used an infrared (IR) sensor to remotely detect and record 2D IR images during excitation by periodically modulated laser radiation [176].

### 4.6. Optical Techniques and Methods

Clinical settings of CT, MRI, PET and ultrasonography cannot reach enough resolution to visualise microcirculation in the CAM. Besides, the contrast agent (or radiotracer) injection may cause unwanted effects [177]. 

However, optical techniques may be advantageous by enabling high-resolution in vivo blood flow imaging of CAM vessels. Blood has a very typical reactive spectrum of light, and important parameters, including blood flow, volume, flow velocity, blood perfusion and the structure of the vessel network in the CAM, can be obtained by optical imaging techniques.

A few optical methods have been developed to provide information about the blood movement, quantified blood flow and perfusion in CAM assays. These innovative applications are mainly derived from three directions: (1) the optical coherence tomography (OCT) principle, (2) the laser Doppler principle and (3) the laser speckle principle.

#### 4.6.1. Optical Coherence Tomography (OCT) Principle

The OCT technique is for non-invasive cross-sectional imaging in biomedical systems. Low-coherence interferometry is utilised to produce 2D images of optical scattering from microstructures of internal tissues. OCT has spatial resolutions longitudinally and laterally in a few micrometres. OCT is analogous to pulse-echo in ultrasonic imaging [178], except that it uses light to replace sound [179]. Cross-sectional images from different depths are extracted by coherence gating, whereby 3D imaging is built from 2D data [180].

However, OCT is not eligible for blood flow imaging due to the interference of the internal tissue background. Hence, optical Doppler tomography (ODT), which integrates the Doppler principle, i.e., Doppler flowmetry, into OCT, was developed to measure the blood flow velocity in biomedical systems. In addition, both blood microcirculation and tissue structures surrounding the vessel can be imaged in vivo non-invasively due to the high resolution of ODT [181]. The blood vessel wall, chorion membrane and yolk sac membrane of the CAM and embryo are depicted in tomographic structural images. In ODT velocity images, blood moving at different velocities appears distinctive in contrast to dark static regions in the CAM. The blood flow velocity is maximum at the vessel centre, decreasing gradually towards the vessel wall, with a velocity resolution of 100 µm/s. The ODT probes into tissues to a depth of about 1–2 mm. 

A Doppler variance optical coherence tomography (DVOCT) method was developed to apply to tumour vasculature imaging. A glioma F98 tumour spheroid was tested on the CAM, and 3D mapping of the tumour was carried out. Both the microstructure and the microvasculature of the tumour were visualised clearly. A dense vascular network of capillaries up to 10 μm in diameter was visualised from the top view of the DVOCT image with a spatial resolution of 3.5 μm and an average penetration depth of 1.5 mm. As a real-time imaging system, DVOCT has the potential to assess tumour vasculature as a non-invasive technique in clinical and preclinical settings [177].

#### 4.6.2. Laser Doppler Principle

Doppler optical theory was first demonstrated to measure the red blood cell (RBC) concentration and blood flow velocity four decades ago. Since then, laser Doppler instruments have been developed [182]. With this approach, one part of the light is scattered by static tissue and remains at its incoming frequency, whereas the other part of the light is scattered by moving RBCs and reflected to a detector, but its frequency shifts due to the Doppler effect. The mixture (interference) of these different frequencies at the detector leads to a beat frequency equal to the Doppler shift between the two light parts. Because the scattering light in tissue is virtually random, such photons have different Doppler shifts. Thus a Doppler frequency spectrum is generated and detected by the detector, which is converted into the power spectrum of the current fluctuations. The power spectrum reflects information about the blood perfusion. Through signal processing, an RBC flux is expressed by a combination between RBC concentration and velocity. Doppler technology has huge publication records due to its maturity and wide applications [183]. 

Laser Doppler perfusion imaging (LDPI) with an area scanning beam represents a recent advance in laser Doppler technology [184]. A CAM-LDPI platform was established to assess drug absorption [185]. Vasoactive drugs were chosen, including caffeine, propranolol, glucagon, theophylline, glyceryl trinitrate (GTN), etc., and common solvents. They were applied on the CAM surface, and the blood perfusion was quantitatively measured by LDPI in response to different concentrations of solvents and drugs. Perfusion is a parameter related to the speed of multiple numbers of RBCs [186], and the CAM is useful for the assessment of drug absorption by using LDPI to measure blood perfusion [184]. New generations of commercial LDPI work with rasters on a line of pixels in parallel at a single time, thereby substantially reducing the acquisition time of single images.

A laser Doppler anemometer (LDA) and signal analysis method were developed to measure the absolute blood flow velocity in animal and human near-surface arterioles and venules [187]. The vessels of the CAM at EDs 9–13 with a diameter of 111–229 μm lying at a depth of 70–200 μm were measured. The study found out how the LDA works, and what considerably influences its measuring capability are periodic and aperiodic blood flow pulsations [187].

Another technology, a complementary metal-oxide semiconductor (CMOS) acquires data from all points through a strong powered expanded laser beam at the same time. Temporal resolution is improved significantly: just 2–10 s are needed for acquisition and display of a 256 × 256–pixel image, instead of 4 min by common point scanning LDPI [183].

Although LDPI is a standard method for optical measurement of blood perfusion and has been improved significantly, it is still limited by low resolution and long measurement times. Therefore, a new analysis technique, laser speckle perfusion imaging (LSPI), was developed, which can create prompt, high-resolution perfusion images [184].

#### 4.6.3. Laser Speckle Principle

The speckle imaging principle was initially exploited for imaging retinal blood flow in the early 1980s [188]. Digital laser speckle imaging is a novel technology, and the first commercial product, full-field laser perfusion imager (FLPI), was introduced in 2007 by Moor Instruments.

Illuminating tissue with coherent light induces a random interference pattern, named speckle. The speckle is formed by constructive and destructive interferences, which are caused by variable lengths of travelling light reverting to the detector due to different pathways traversing in the object and irregular surfaces [189].

Any motion of the particles in tissue leads to speckle pattern changes. If there is flow in the region of interest (ROI), the speckle pattern undergoes decorrelation (or blur) and the intensity in ROI decreases. Thus, the level of decorrelation depends on the speed and volume of the RBCs in the tissue area. Under this logic, low intensity is relevant to a high amount of blood flow (because the light is scattered by moving RBCs), and conversely, high intensity expresses low blood flow. The speckle size is determined by the aperture size of the imager alone. Relative to the correlation time of intensity fluctuations, the detector integration time is sufficiently short. For imaging blood perfusion in the skin, the integration time is set to between 5 and 20 ms in a typical device [183]. By analysing intensity fluctuations using computer software and algorithms, real-time graphs and other temporal and spatial information about blood perfusion in the tissue can be obtained. Because the speckle pattern reflects the dynamics of the scatters, speckle imaging is very beneficial for studying the fluid within turbid tissues in biomedical applications [189].

LSPI can response to changes in both blood flow velocity (0–800 L/min, 1% concentration) and RBC concentration (0.1%–5%, 200 L/min) and provide a quicker response time and higher spatial resolution, which is more advantageous to LDI for certain uses. Therefore, LSPI and its commercial instrument were validated for measuring tissue blood perfusion [184]. However, they are still rather new and not yet mature [183]. People are still striving to explore new applications of LSPI in preclinical tests, for example in mice [190,191,192], rabbits [193,194] and even human skin [195].

Concerning the LSPI application on the CAM, only a few efforts have been documented. In 2005, when a commercial LSPI instrument had not been released, a team used a customised speckle imaging device and software to monitor changes in the vascular structure and blood flow after photodynamic therapy (PDT) in the CAM at EDs 10–13. The laser was used to stimulate PDT and also to function as the coherent light source for LSPI [91]. At EDs 9–11, tumour cells from a lung metastasis human salivary adenoid cystic carcinoma cell line were inoculated on the CAM. At ED 11 or ED 12, a photosensitiser of pyropheophorbide acid (Pyro-Acid) solution was applied topically on the vessel-rich position surrounding the tumour graft on the CAM. The treatment lasted 30 min before laser irradiation for 15 min. The blood velocity changes at the treated area were recorded by speckle imaging at 0, 3, 6, 9, 12 and 15 min after PDT. The results showed that the blood velocity in vessels slowed down (small vessels decreased to approximately 10% and big vessels decreased to about 25% compared to the beginning) due to vascular damage such as vascular constriction, blood stasis and coagulation. The results suggested that speckle imaging is effective to assess the PDT efficacy in peripheral vessel damage of the tumour through monitoring changes in the vascular structure and blood flow.

However, this trial had some deficiencies: (1) the measurement was rough, so the extent of vessel changes and blood flow perfusion needs a precise standard; (2) only a basic equation was used in the calculation, but optical properties of scatters (scatter size and multiple scattering) were not considered; (3) peripheral vessels of the tumour on the CAM were measured, but intratumoral vasculature was not considered; (4) all experiments were in vitro (ex ovo); and (5) only a customised device was used. Due to the lack of a standard instrument, speckle imaging is still difficult to be popularised in preclinical assays and clinical applications.

More recently, the impact of the optical properties of scatters was noted and experimentally investigated, which is relevant to the quantification of the inverse relation between decorrelation time (*τ*c) and blood flow velocity (*V*), i.e., 1/*τ*c = *αV*. A sidestream dark-field–laser speckle contrast imaging (SDF-LSCI) device was designed to record the specific vessels of the CAM at ED 9, grown ex ovo. *V* (by SDF) and *τ*c (by LSCI) of the same vessels were independently estimated. Thus, a practical speckle model, considering multiple scattering (the influence on α), was summarised, and a blood flow velocity map was generated. The vascularised CAM can be an intermediate calibration model [196].

Non-invasive imaging methods are still not optimal to evaluate blood flow, blood volume and vascular change and cannot detect functionally structural changes in deep tissues [157]. However, due to its uniqueness, the CAM could be a good platform to foster more precise methods and tools to assess changes in normal tissue vasculature and tumour blood vessels before and after treatment with different therapies, and thus to conduct pharmacological research.

### 4.7. Artificial Intelligence (AI) Techniques and Algorithms

Chick embryos are also used for virus culture during the vaccine preparation process for common industrial production. The quality of embryos crucially impacts the quality of the vaccine. Therefore, any dead, broken, contaminated and weak embryos unsuitable for vaccine preparation, and for other CAM studies as well, need to be eliminated. The morphology and features of blood vessels in the CAM during certain ED periods provide a feasible tool for the detection of unsuitable embryos.

Machine inspection is a popular approach to determining the viability of embryos by extracting imaging information about the major blood vessels of the embryo. Several machine vision methods are in use, which perform image enhancement, segmentation and classification. They mainly focus on the major artery and vein. The limitations of image acquisition conditions, image quality and complexity of specific embryos largely influence the quality of classification [197]. 

Therefore, improved algorithms have been successfully applied to analyse and reconstruct images. The results demonstrate that the improved algorithms have a much higher anti-noise capacity and can greatly improve the quality of image reconstruction [174].

Further innovation has transited to systemic AI methods, which consolidate advanced image reconstruction and analysis. In recent years, as typical deep learning methods, convolutional neural networks (CNNs) have shown promising performance in image classification. A CNN refers to a multi-layer, self-supervised learning perceptron. It is composed of a data layer, a multiple alternating convolution layer, a pooling layer, a fully connected layer and an output layer [198]. Several CNNs have been proposed for embryo activity or viability detection based on many existing models. In 2017, Geng et al. proposed a deep-learning-based classification method for embryo viability detection using a unique CNN structure. It greatly improved the detection accuracy up to 99.5% [197].

AI methods can sufficiently achieve viability detection, but they are not effective for weak embryo detection. In 2020, based on previous CNN models, Chen et al. proposed a weak embryo detection method based on the multiscale feature fusion convolutional neural Weak Embryo Detection Network (WEDNet) [199]. The proposed residual multiscale fire block (RMFB) to extract multiscale features from egg embryo images and feature fusion performance could effectively use the overall colour information about the image and blood vessel information at different scales. The detection accuracy of WEDNet, constructing through the cascade of RMFB modules, could reach 99.35% [199]. 

Through AI techniques and algorithms, a completely non-invasive method to extract and analyse blood vessel information about chick embryos has been introduced. Vaccine production as well as CAM studies can be improved by good embryo detection, classification and selection.

## Figures and Tables

**Table 1:** Circulating blood volume (mL) in chick embryo on different embryonic days (EDs).

ED	4	5	6	7	8	9	10	12	14	16	18
Blood Volume (mL)	0.04	0.10	0.17	0.26	0.37	0.51	0.68	1.15	2.15	3.13	2.58

**Table 2:** Representative growth factors for angiogenesis in the CAM.

Growth Factors	Description	Functions	Ref.
VEGF	Vascular endothelial growth factor	To exert vascular permeability and endothelial cell recruitment	[37]
FGF	Fibroblast growth factor	To elicit fibrocyte proliferation	[11,39,40]
PDGF	Platelet-derived growth factor	To stimulate vascular stability	[38]
ANG	Angiopoietin	To act on endothelial sprouting, vessel wall remodelling and mural cell recruitment	[37]
HGF	Hepatocyte growth factor	As a cytokine to stimulate proliferation and morphogenesis of epithelia	[37]
HIF	Hypoxia-inducible factor	To induce expression of VEGF and its receptors	[14,37]
Endostain	A proteolytic fragment of collagen XVIII (a component of the basement membrane)	To act as an endogenous anti-angiogenic molecule	[37]

**Table 3:** Methods to apply pro-angiogenic agents on the CAM.

Model in Technical Materials	Ref.
Filter disks	[74]
Culture coverslide glasses	[75]
Inert synthetic polymers: Elvax 40 (ethylene-vinyl acetate copolymer)	[76]
Hydron (a poly-2-hydroxyethylmethacrylate polymer)	[76]
Methylcellulose disks	[77]
Alginate pellet	[78]
Gelatin sponges	[79]
Cross-linked collagen hydrolysate	[80]
Cross-linked collagen matrices	[81]
Cross-linked and heparinised collagen matrices	[81]
Fibrin matrices	[71]
Poly(D,L-lactic acid) (PLA)	[82]
Poly(ethylene glycol) (PEG)	[83]
Modified Matrigel mixtures	[84]
Paraffin and plastic embedding	[85]

**Table 4:** Direct and indirect angiogenesis inhibitors.

Direct	Indirect
Angiostatin, bevacizumab (Avastin) Arresten, canstatin, combrestatin Endostatin, thrombospondin Tumstatin, methoxyestradiol, vitaxin	Targeting EGF-receptor tyrosine kinase Sunitinib Targeting the VEGF receptor Targeting the PDGF receptor Targeting Erb-B2 Receptor Tyrosine Kinase 2 (ERBB-2) Targeting the interferon alpha receptor *

* Interferon alpha can be considered both a direct angiogenesis inhibitor because it inhibits endothelial migration and an indirect angiogenesis inhibitor because it inhibits synthesis of FGF by tumour cells [57,89].

**Table 5:** Drug assays for tumour grafts or metastasis on a CAM platform.

Drugs	Type of Tumour	Ref.
Hydrophobic derivatives of boswellic acid	PC-3 human prostate cancer	[111]
Monoclonal antibodies (anti-GD2) or Asn-Gly-Arg (NGR) peptides incorporated in liposomes	Neuroblastoma	[112]
Photosensitiser Ce6	Bladder cancer	[113]
Bis(methoxyethyl)-di-n-propylporphycene (BEPPn)	RR 1022, epithelial cells from Rous sarcoma virus (RSV)	[114]
Photosensitising drug: methylene blue (MB)	Human malignant ovarian adenocarcinoma	[92]
Sema3C	Glioblastoma (U87 MG cells)	[115]
Streptokinase and gemcitabine	Lewis lung carcinoma	[103]
Neutralising antibody against protein A	Ovarian cancer	[116]
Doxorubicin in nanoparticles	Ovarian cancer	[53]

**Table 6:** Vascular-disrupting agents (VDAs) and developmental status.

VDAs Name	Company	Stage of Clinical Development	Ref.
**Tubulin Binding**			
CA4P (fosbretabulin)	Mateon Therapeutics	Phase 2/3	[3,49]
C118P	Sanhome Pharmaceutical	Phase 2	[128]
Combretastatin A4 gold derivative	University Bayreuth	New formulation under research	[129]
Deoxypodophyllotoxin (DPT)	China Pharmaceutical University	New formulation under research	[130]
NPI-2358 (plinabulin)	BeyondSpring	Phase 3	[3]
BNC105P	Bionomics	Phase 2	[3]
EPC2407 (crolibulin)	Immune Pharmaceuticals	Phase 1/2	[127]
OXi4503 (CA1P)	Mateon Therapeutics	Phase 1/2	[3]
CKD-516	Chong Kun Dang Pharmaceutical	Phase 1	[3,131]
MN-029	Medicinova	Phase 1	[3]
ENMD-1198	EntreMed	Phase 1	[132]
C9	Shanghai Institute of Materia Medica, Chinese Academy of Sciences	New formulation under research	[133]
Phenyl-3-(2-chloroethyl) urea (CEU)	IMOTEP Inc.	New formulation under research	[134]
BPR0C261	National Health Research Institutes, Taiwan	New formulation under research	[135]
BPR0L075	National Health Research Institutes, Taiwan	New formulation under research	[136]
IMC-038525	ImClone Systems	New formulation under research	[137]
ABT-751	Abbott	Phase 2	[3]
AVE8062 (ombrabulin)	Sanofi-Aventis	Development terminated	[3]
CYT997 (lexibulin)	Gilead	Development terminated	[3]
Dolastatin-10	Marine Biotech	Phase 2	[3]
MPC-6827 (verubulin, Azixa)	Myrexis	Phase 2	[3]
TZT-1027 (soblidotin)	Daiichi-Sankyo	Phase 2	[3]
ZD6126 (ANG453)	AstraZeneca	Phase 2	[3]
**Flavonoids**			
ASA404 or DMXAA	Antisoma	Phase 3	[3]

**Table 7:** Studies of VDAs on the CAM.

VDAs	Test Methods	Ref.
Combretastatin A4-phosphate (CA4P)	Topical paper	[144]
CA4 gold derivative	Topical thin silicon foil	[129]
Deoxypodophyllotoxin (DPT)	Topical filter paper disks	[130]
C9	Topical	[133]
Verubulin (MPC-6827)	Topical silicon foil ring	[145]
Phenyl-3-(2 chloroethyl) urea (CEU)	Intravenous injection	[134]

**Table 8:** Advantages and limitations of the CAM as a screening platform.

	CAM Features	Research Methodology
**Advantages**	Small size and easy to handle	Reproducibility and reliability
	Contains rich nutrients and intensive angiogenesis capacity	Great accessibility
	Rapid vascular growth	Rapid screening platform
	Necessary organs isolated within the CAM, in vivo	Broad imaging modalities available
	High embryo survival rate	No mobility of animal
	Complete circulatory system (intravascular delivery of drugs)	Cost-effectiveness
	Naturally immune deficient, different tissues and species transplantations without immune responses	Topical and intravascular administration of drugs
	No requirement for animal protocol approval, less animal welfare burden	
**Limitations**	Chicken origin of the assay limiting availability of reagents	Very thin and fragile, careful manipulation required
	CAM under rapid change	Complex choice of protocols available
	Developed vascular network difficult to be distinguished from new capillaries	Difficult real-time monitoring
	Sensitive to environmental factors (temperature, oxygen tension)	Short observation time after treatment
	Differences in drug metabolism with mammals	Post-grafting non-specific inflammatory reactions after ED 15
	False angiogenesis due to dust and other irritants	Oral drug administration not feasible
